# A Comparative Analysis of Reactive Müller Glia Gene Expression After Light Damage and microRNA-Depleted Müller Glia—Focus on microRNAs

**DOI:** 10.3389/fcell.2020.620459

**Published:** 2021-01-26

**Authors:** Seoyoung Kang, Daniel Larbi, Monica Andrade, Sara Reardon, Thomas A. Reh, Stefanie G. Wohl

**Affiliations:** ^1^Department of Biological and Vision Sciences, College of Optometry, The State University of New York, New York, NY, United States; ^2^Department of Biological Structure, School of Medicine, University of Washington, Seattle, WA, United States

**Keywords:** retinal degeneration, gliosis, injury, miR-125, let-7, stress response, Maff, Gadd45b

## Abstract

Müller glia (MG) are the predominant glia in the neural retina and become reactive after injury or in disease. microRNAs (miRNAs) are translational repressors that regulate a variety of processes during development and are required for MG function. However, no data is available about the MG miRNAs in reactive gliosis. Therefore, in this study, we aimed to profile miRNAs and mRNAs in reactive MG 7 days after light damage. Light damage was performed for 8 h at 10,000 lux; this leads to rapid neuronal loss and strong MG reactivity. miRNAs were profiled using the Nanostring platform, gene expression analysis was conducted via microarray. We compared the light damage dataset with the dataset of Dicer deleted MG in order to find similarities and differences. We found: (1) The vast majority of MG miRNAs declined in reactive MG 7 days after light damage. (2) Only four miRNAs increased after light damage, which included miR-124. (3) The top 10 genes found upregulated in reactive MG after light damage include *Gfap, Serpina3n, Ednrb* and *Cxcl10*. (4) The miRNA decrease in reactive MG 7 days after injury resembles the profile of Dicer-depleted MG after one month. (5) The comparison of both mRNA expression datasets (light damage and Dicer-cKO) showed 1,502 genes were expressed under both conditions, with *Maff* , *Egr2, Gadd45b*, and *Atf3* as top upregulated candidates. (6) The DIANA-TarBase v.8 miRNA:RNA interaction tool showed that three miRNAs were found to be present in all networks, i.e., after light damage, and in the combined data set; these were miR-125b-5p, let-7b and let-7c. Taken together, results show there is an overlap of gene regulatory events that occur in reactive MG after light damage (direct damage of neurons) and miRNA-depleted MG (Dicer-cKO), two very different paradigms. This suggests that MG miRNAs play an important role in a ubiquitous MG stress response and manipulating these miRNAs could be a first step to attenuate gliosis.

## Introduction

Müller glia (MG) are the predominant glia in the neural retina and fulfill, similar to astrocytes in the brain, a variety of crucial functions to ensure retinal homeostasis and proper neuronal function. These functions include neurotransmitter uptake and recycling via specific enzymes such as glutamine synthetase (GS), nourishment of neurons by providing lactate, tissue stabilization and structural support by forming the inner and outer limiting membrane, maintenance of the blood retinal barrier (BRB), and tissue protection (Reichenbach et al., 1993; Newman and Reichenbach, 1996; Bringmann et al., 2000, 2006; Bringmann and Reichenbach, 2001; Kuhrt et al., 2008; Reichenbach and Bringmann, 2013). The MG response to retinal injury is known as gliosis. There are two kinds of MG gliosis, an unspecific and a specific response. The unspecific response is characterized by hypertrophy (swelling) and the upregulation of the intermediate filament GFAP, which is considered a marker of reactivity. The specific response to neuronal damage includes the downregulation of the enzyme glutamine synthetase in models of massive and rapid photoreceptor loss such as light damage, or retinitis pigmentosa (Bringmann et al., 2006). The gliotic response requires substantial changes in gene expression and it is currently not known whether miRNAs play a role in this process.

miRNAs are short (18–24 nucleotide long) non-coding RNAs and act as transcriptional repressors. They are transcribed as a primary molecule that is processed by the microprocessor complex to a precursor miRNA, which is cleaved by the endoribonuclease Dicer1 into the mature and functional form. Mature miRNAs then bind predominantly (but not exclusively) on the 3′ untranslated region (UTR) of a messenger RNA (mRNA) and (1) inhibit translation into protein (imperfect complementary binding) or (2) induce mRNA decay (perfect complementary binding). This occurs in the RNA-induced silencing complex (RISC) in which the miRNA is bound to a protein called Argonaute 2 (Ago2) (Gurtan and Sharp, 2013; Roberts, 2015). miRNAs play important roles during retinal development by regulating cell division, cell maturation and cell fate specification. Because of this regulatory impact, miRNAs play a detrimental role in cancer and other diseases including retinal diseases (Sundermeier and Palczewski, 2012; Zuzic et al., 2019), but we are just beginning to understand the critical miRNAs and their mRNA targets in the retina.

We previously profiled the microRNAs (miRNAs) expressed in MG and studied their impact on MG function by deleting the endoribonuclease Dicer1 (Dicer-cKO^MG^), the enzyme that generates mature miRNAs. In the Dicer-cKO^MG^ retinas, we observed a progressive retinal disorganization and a loss in rod photoreceptors and retinal function (Wohl et al., 2017). Interestingly, the deletion of Dicer1 in MG did not cause an upregulation of GFAP, the hallmark of gliosis. Moreover, the loss of rod photoreceptors and retinal disorganization had some similarities to the phenotype observed in end-stage retinitis pigmentosa. Since this phenotype displayed similarities to diseases characterized by massive photoreceptor loss, although no neuron was primarily affected, we aimed to profile the MG miRNAs and mRNA expression after light damage. Light damage is a commonly used model to study retinal degeneration that resembles age-related macular degeneration (AMD) or retinitis pigmentosa (Winkler et al., 1999; Beatty et al., 2000; Wenzel et al., 2001, 2005; Chen et al., 2004; Samardzija et al., 2006; Grimm and Reme, 2013; Luu et al., 2020). The light damage model is a very flexible model and can be altered with regard to light intensity (2,000–100,000 lux) and duration (minutes–days). Nevertheless, it is a very robust and well-studied model (Burns and Robles, 1990; de Raad et al., 1996; Chen et al., 2004; Rattner and Nathans, 2005; Gosbell et al., 2006; Ueki et al., 2008; Natoli et al., 2010; Ueki and Reh, 2012).

To our knowledge, there are no reports available about the miRNA profile of reactive MG. Therefore, in this study, we aimed to identify the miRNAs that might regulate genes involved in gliosis by performing global comparisons of reactive MG 7 days after light damage and MG after Dicer deletion (loss of miRNAs). We light damaged adult mice and analyzed neuronal loss and glial response after 1 week, when the vast majority of photoreceptors were lost, and MG were reactive (gliosis). miRNAs from light damaged MG were profiled using Nanostring technologies. We found that the vast majority of miRNAs highly expressed in MG declined after light damage, similar to what we found in the Dicer-cKO^MG^. We used microarray for gene expression analysis in order to identify the genes that are upregulated the most 1 week after light damage and could represent potential miRNA targets. Most genes were associated with cell death and inflammatory response. Since the miRNA was similar, we compared that dataset with that from Dicer-cKO^MG^. Thousand five hundred and two genes were expressed in both datasets, with *Atf3, Egr2, Maff*, and *Gadd45b* as top candidates, genes involved in stress response. The identified miRNAs, potentially targeting these genes, are miR-125b-5p, let-7c, and let-7d. This data suggests that independent from extrinsic influences, a common intrinsic glial stress program appears to be activated that is directed by MG miRNAs. This is particularly of importance for understanding and attenuating gliosis. MG miRNAs are therefore potential promising tools for counteracting glial alterations and this study is the first attempt to narrow down the list of candidates for subsequent downstream experiments.

## Materials and Methods

### Animals and Cre Induction

All mice were housed at the State University of New York, College of Optometry and used in accordance with the Institutional Animal Care and Use Committee approved protocols (IACUC). *Rlbp1-creERT2* mice (obtained from Dr. Edward Levine, S129 background) were crossed to *R26-stop-flox-CAG-tdTomato* mice (Jackson Labs, also known as Ai14, #007908) and will be henceforth referred to as *RlbpCreER: stop*^*f*/*f*^*-tdTomato* or wild type (wt). For light damage mice, Rlbp1-creERT:tdTomato mice were crossed to the albino Swiss Webster mouse (CSW 024, Charles River Laboratories) that carries the RPE65^450Leu^ gene (confirmed by genotyping). In addition, the Hes5-GFP mouse (Basak and Taylor, 2007) was used as another MG-specific reporter mouse established in the lab (Nelson et al., 2011). The Hes5 mouse (S129 background) was also crossed to the Swiss Webser mouse. Genotyping was done using the primers listed in Supplementary Table 1. For the detection of RPE65 variants, a subsequent digest of the PCR product with the restriction enzyme MwoI was performed for 2 h at 37°C. Tamoxifen (Sigma, St. Louis, MO) was administered intraperitoneally at 75 mg/kg in corn oil for four consecutive days in adult mice (2–3 months of age) to initiate the recombination of the floxed alleles.

### Light Damage

Mice were exposed to diffuse, cool, white light (bulbs are located on top of the cage). Food and water were placed in the cage to avoid blocking light exposure. Luminance (~10,000 lux) was measured on the cage floor using a light meter. Mice were exposed to the light for 8 h and returned under normal lighting (12 h on/12 h off cyclic light) for recovery. Analysis was performed 7 days after light damage (LD).

### Fluorescence Activated Cell Sorting (FACS)

All retinas were checked for successful recombination under the fluorescence microscope before every sort. For each sort, about 6–10 retinas were pooled and dissociated in DNase/Papain (75 μl/ 750 μl, respectively, Worthington) for 20 min at 37°C on the shaker, triturated, mixed with Ovomucoid (750 μl), centrifuged for 10 min at 300 × g and resuspended in 800 μl DNase/ Ovomucoid/ Neurobasal solution (1: 1: 10, respectively, Gibco) per retina. Cells were filtered through a 35 μm filter, sorted using an 85 micron nozzle, and collected into two chilled tubes. Cell sorts were performed using BD Aria III cell sorter (BD Bioscience). Debris was excluded from the sort and only all events in gate P1 were sorted (Supplementary Figure 1A). Cells with the brightest fluorescence were found in gate P3 (“positives,” MG fraction), cells with no fluorescence in gate P2 (“negatives,” neuronal fraction, Supplementary Figure 1A′), everything in between was excluded. Gating settings were kept throughout all sorts for undamaged and damaged retinas. The fraction of MG comprised about 1.7% Supplementary Figures 1A″, *A*^‴^). Samples were collected in FBS-coated tubes containing Neurobasal medium. After collection, the tdTomato^+^ MG fraction (P3) and the tdTomato^−^ fraction (P2) was post-sorted to validate purity (Supplementary Figures 1B–B″,C–C″). Cells were spun for 10 min at 300 × g at 4°C, the pellet was homogenized in Qiazol (Qiagen) and stored at −80°C.

### RNA Purification, miRNA, and mRNA Profiling

For miRNA profiling, the sorts of 44 light damaged retinas were pooled for RNA purification. RNA was extracted and purified with a miRNeasy Micro Kit in accordance with manufacturer's instructions (Qiagen). NanoString nCounter was used for miRNA expression analysis. Two hundred ng total RNA per sample (33 ng/μl) was submitted for NanoString analysis. NanoString data was analyzed using nSolver 4.0 software. The data represents counts of molecules normalized against 4 housekeeping genes (β*-actin, GAPDH, Rpl19*, and *B2m*), 8 negative controls, and 6 positive controls that were run with the samples. miRNA data after Dicer-cKO and for wild type MG was published before (Wohl et al., 2017), is available at GEO (GSE 103098). Raw data was *de-novo* normalized and analyzed together with the light damaged data. For Microarray, 12 retinas were used for controls and 10 retinas for light damage, FACS-purified, the RNA isolated and run on the Mouse Gene 1.0 ST microarray (Affymetrix) according to manufacturer's guidelines. The RIN numbers for the samples ranged from 8 to 10 with a mean of 8.98. The microarray data was normalized and analyzed with Affymetrix Power Tools software and TM4 Multi-Experiment Viewer software. RNA-Seq data of pigmented adult wild type MG was published before (Wohl et al., 2017), is available at SRA (NBCI, SRP115835) and was used for gene expression comparisons. Also, the datasets of control FACS purified MG and MG 36h after light damage, as well as 48h after NMDA damage from Hoang et al. (2020) were used for gene expression comparisons.

### Fixation, Sectioning, and Immunofluorescent Labeling

Mouse eyes were fixed in 4% PFA for 30–60 min, treated with 30% sucrose in PBS overnight, embedded in O.C.T. embedding medium, and cross sectioned in 12 μm thick sections. For immunofluorescent staining, cells were incubated in blocking solution (5% milk block: 2.5 g non-fat milk powder in 50 mL PBS; with 0.5% Triton-X100) for 1 h at RT. Sections were incubated with primary antibodies (Supplementary Table 2) in 5% milk block overnight, secondary antibodies (Invitrogen/Molecular Probes, and Jackson ImmunoResearch, 1:500–1,000) for 1 h at RT and counterstained with 4′,6-diamidino-2-phenylindole (DAPI, Sigma, 1:1,000).

### Microscopy, Cell Counts, and Statistical Analysis

Live imaging was performed using Zeiss Observer D1 with Axio-Cam. Fixed cells were analyzed by Olympus FV1000 or Zeiss LSM 880 confocal microscope as well as Keyence BZX 800 for overview images. For retinal cross sections, two areas/section with 625 μm × 625 μm dimension at 200 × magnification, four optical sections of 2 μm thickness, for two sections per mouse, of at least six light damaged and undamaged controls were counted. For Otx2 cell assessment in the ONL, vertical rows of cells were counted for six different areas per image. Values are expressed as mean ± standard deviation (S.D.). Statistical analyses were performed by Mann-Whitney (U) test for independent samples and the Wilcoxon test for dependent samples. Holm-Bonferroni method was used to correct for multiple comparisons.

### miRNA-Target Interaction Analysis, Ago HITS-CLIP, Gene Ontology

For miRNA-mRNA interaction analysis, DIANA-TarBase v.8 (https://carolina.imis.athena-innovation.gr/diana_tools) was used, a database of experimentally supported miRNA:mRNA interactions (Karagkouni et al., 2018). It integrates information on cell-type specific miRNA–gene regulation, while thousands of miRNA-binding locations are reported. It is the first database indexing more than 1 million entries, corresponding to ~670 000 unique miRNA-target pairs. The interactions are supported by >33 experimental methodologies, applied to ~600 cell types/tissues under ~451 experimental conditions (Karagkouni et al., 2018). All gene names were inserted and miRNA candidates that were found in MG, selected and depicted. For miRNApathway analysis DIANA-mirPath v.3 was used. DIANA-mirPath can utilize predicted miRNA targets (in CDS or 3′-UTR regions) provided by the DIANA-microT-CDS algorithm or even experimentally validated miRNA interactions derived from DIANA-TarBase (Vlachos et al., 2015). STarMirDB (http://sfold.wadsworth.org/starmirDB.php) was used to find and visualize the individual miRNA binding sites in mRNA target 3′ UTRs (Rennie et al., 2016). The database allows a fast search of pre-computed results that were enriched for miRNA binding sites identified from CLIP data. The transcriptome-scale predictions results are categorized into seed and seedless sites in 3′ UTR, CDS and 5′UTR, and provide a list of sequences, thermodynamic and target structural features. A logistic probability model was used as a measure of confidence of the site being a miRNA binding site (threshold 0.5–0.7).

Ago HITS-CLIP [Argonoute2 **hi**gh-**t**hroughput **s**equencing of RNAs isolated by **c**ross**l**inking **i**mmuno**p**recipitation developed in the Darnell lab (Chi et al., 2009)] data for whole undamaged and light damaged retinas; generated in the Natoli lab (available at BioRxiv), was used to evaluate whether MG miRNAs and mRNAs are Ago2 bound (Chu-Tan et al., 2020).

For mRNA gene ontology, ShinyGO v0.61 (http://bioinformatics.sdstate.edu/go/) was used. Genes were inserted, GO Biological Process selected, *P*-value cutoff (FDR, false discovery rate) of 0.05 chosen and the 20 most significant terms selected to be depicted.

## Results

### Light Damage Leads to Neuronal Loss and Müller Glia Activation

**Figure 1:**
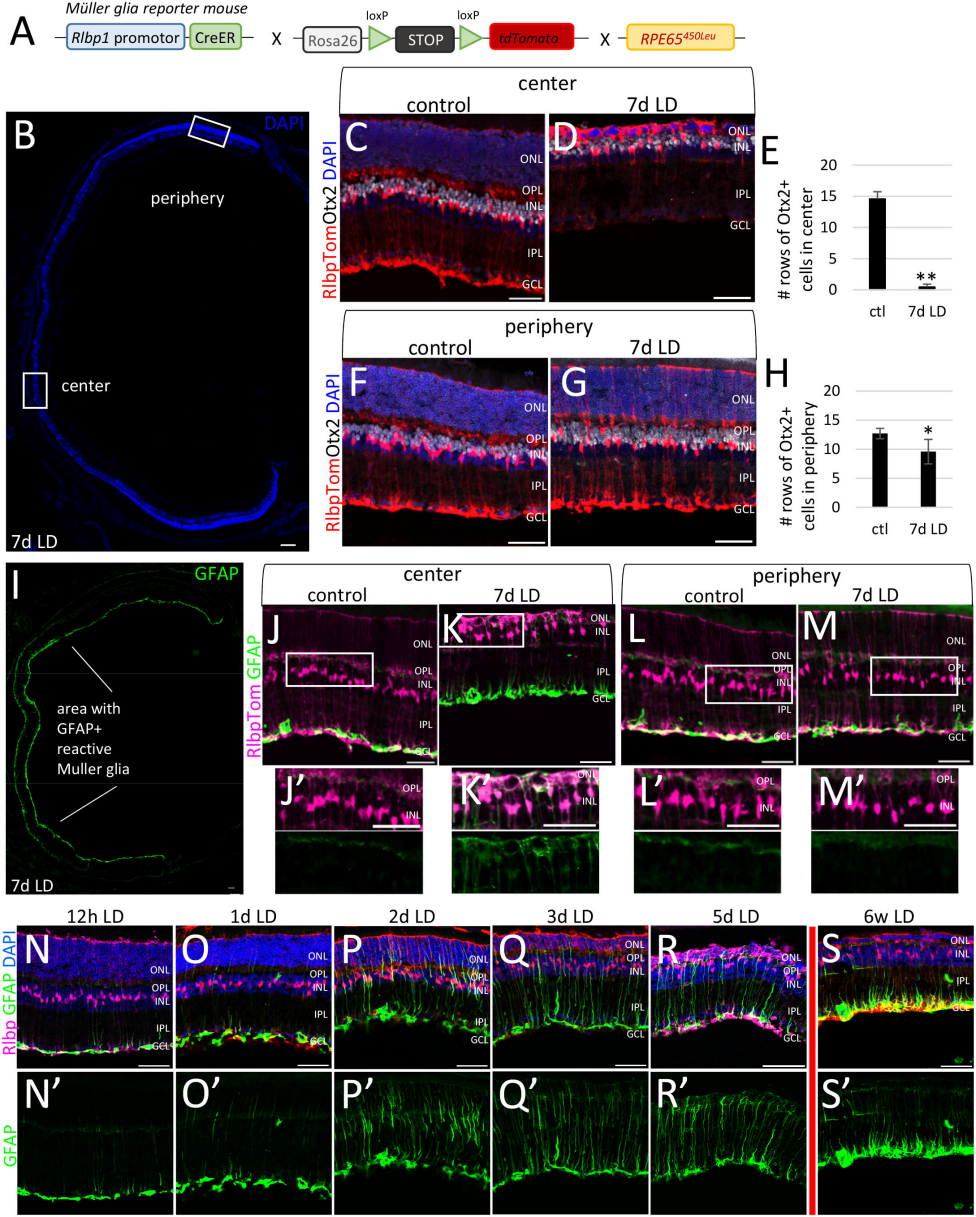
Neuronal loss and gliosis in the central and peripheral retina after light damage. **(A)** Schematic of the *RlbpCreER: stop*^*f*/*f*^*-tdTomato: RPE65*^450*Leu*^ mouse. **(B)** Cross section of DAPI-labeled retina 7 days after light damage with regions of analysis. **(C–G)** Immunofluorescence labeling for tdTomato (MG), Otx2, and DAPI nuclear staining of center **(C,D)** and peripheral areas **(F,G)** of undamaged and light damaged (7d LD) retinal sections. **(E)** Number of vertical cell rows of Otx2+ photoreceptors in the ONL of the central retina in controls and 7 days after light damage (7d LD). **(H)** Number of vertical cell rows of Otx2+ photoreceptors in the ONL of the peripheral retina in controls and 7 days after light damage (7d LD). **(I–M/M′)** Immunofluorescence labeling for tdTomato (MG) and GFAP of an entire retinal cross section **(I)**, in the central **(J/J′-K/K′)** and peripheral areas **(L/L′-M/M′)** of undamaged and light damaged (7d LD) retinal sections. **(N/N′-S/S′)** Immunofluorescence labeling for tdTomato (MG), GFAP, and DAPI nuclear staining of the center retina 12 h, 1, 2, 3, and 5 days as well as 6 weeks after light damage (LD). Scale bar in B, I 100 μm, in C-S 50 μm. Significant differences are indicated: *p < 0.05, **p < 0.01, *U*-test, n control = 5, n LD = 7. ONL, outer nuclear layer; OPL, outer plexiform layer; INL, inner nuclear layer; IPL, inner plexiform layer; GCL, ganglion cell layer; LD, light damage.

In order to isolate reactive Müller glia (MG) from light damaged retinas, we crossed the MG reporter mouse with a mouse that has an amino acid variation (leucine instead of methionine) at position 450 in the retinal pigment epithelial protein RPE65, referred as RPE65^450Leu^ mouse (Figure 1A). This variant found in all mouse strains except C57BL/6 (which are commonly used as background mice) has been discovered as a genetic modifier of susceptibility to light-induced damage mice (Danciger et al., 2000; Wenzel et al., 2001, 2005). We aimed to profile reactive adult MG in the acute phase after damage and therefore chose 7 days after damage for analysis. Photoreceptor death begins as early as 24 h (37% of photoreceptors are lost, Supplementary Figures 2A–E). After 7 days, about 90% of photoreceptors (almost entire outer nuclear layer, ONL) were lost in the central retina (Figures 1B–E), while cell death was attenuated in the peripheral retina (Figures 1F–H).

This gradient of neuronal loss from center to periphery was reflected in the MG response. While GFAP protein was strongly upregulated in MG in the central retina (Figures 1I,J/J′-K/K′), MG in the periphery remained GFAP^−^ (Figures 1I,L/L′-M/M′). As we were interested in profiling reactive MG in acute phase of injury, we chose 7 days post injury to have at least 50% reactive MG in the tissue (Figure 1I). GFAP upregulation in the central retina can be detected as early as 1 day after injury and lasts for several weeks (Figures 1N/N′-S/S′).

**Figure 2:**
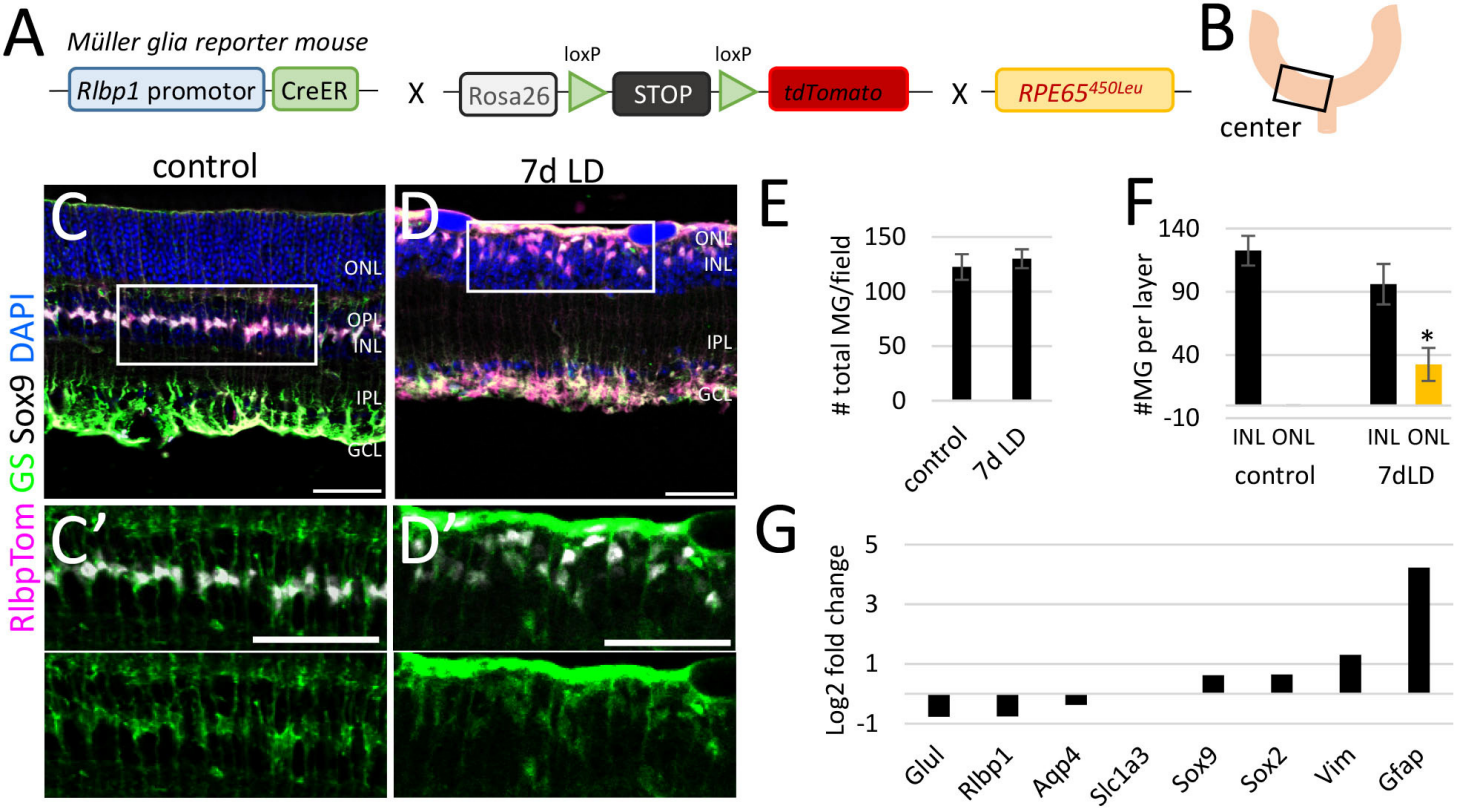
Müller glia number and location in the central retina after light damage. **(A)** Schematic of the *RlbpCreER: stop*^*f*/*f*^*-tdTomato: RPE65*^450*Leu*^ mouse. **(B)** Region of analysis. **(C/C′,D/D′)** Immunofluorescence labeling for tdTomato (MG), glutamine synthetase (GS), Sox9, and DAPI nuclear staining of retinal sections from undamaged controls and 7 days after light damage. **(E)** Number of MG per field in undamaged mice and 7 days after light damage (LD). **(F)** Number of MG in the INL and ONL in undamaged controls and 7 days after LD. **(G)** Expression levels (log_2_ fold change light damage vs. control) of glial genes in undamaged (6 biological replicates, one technical replicate) and damaged retinas (five biological replicates, one technical replicate). Scale bars 50 μm. Significant differences are indicated: *p < 0.05, Wilcoxon-test, n control = 5, n LD = 5. ONL: outer nuclear layer; OPL, outer plexiform layer; INL, inner nuclear layer; IPL, inner plexiform layer; GCL, ganglion cell layer; LD, light damage.

Since there was this substantial neuronal loss resulting in significant retinal thinning in the center retina, we next evaluated aspects of unspecific and specific glial responses due to this neuronal loss. We determined the number of MG cells and their location in the retinal tissue to assess potential proliferation and migration processes. In the center of undamaged control retinas, we found about 120 tdTomato+ MG per field and all were located in the center of the INL, expressing the nuclear marker Sox9 and cytoplasmic enzyme glutamine synthetase (GS, Figures 2A–C/C′,E,F, Supplementary Figures 3A/A′ -E/E′). Seven days after LD we found ~130 tdTomato+ MG expressing Sox9 and GS in the lower INL and in the remaining OPL (1–2 cell layers, Figures 2D/D′–E,F, Supplementary Figures 3F/F′-J/J′) indicating that MG migrated toward the injury side. GS expression in the migrating MG was rather diffuse and could indicate a reduction of GS levels, which has been reported to be a specific MG response in models with massive photoreceptor loss (Bringmann et al., 2009). However, most glial genes were not found to be differently expressed in FACS-purified MG 7 days after light damage, except the intermediate filament GFAP (Figure 2G, Supplementary Table 3). MG comprise only 2–3% of the total retinal cell population (Jeon et al., 1998). Consequently, multiple retinas (biological replicates) need to be pooled for sufficient amounts of RNA from FACS-purified MG. This results in low technical replicate number. To test the robustness of the microarray dataset (6 biological replicates, one technical replicate), we compared the gene expression levels of known glial genes from our microarray dataset (log_2_ of relative expression, fold change 7d light damage vs. control) with RNA-Seq data (fold change 36 h light damage vs. control) by performing a linear regression analysis. This 36 h light damage data set is from a recent global gene expression study [4 technical replicates of FACS-purified MG from pigmented Glast-Cre-GFP reporter mice (Hoang et al., 2020), Supplementary Figure 4A]. This analysis resulted in a high coefficient of determination (*R*^2^) indicating a strong positive correlation of the gene expression patterns of these different methods and different mouse strains (Supplementary Figure 4B). A high correlation of differentially expressed genes of microarrays and RNA-Seq has been reported before (Rao et al., 2018) and was also demonstrated in a white paper from Illumina (Illumina_Inc., 2011). We next analyzed the rigor and robustness of our pooled MG samples and compared our RNA-Seq dataset of wild type RlbpCreERT:tdTomato mice (log_2_ counts per million of adult pigmented wild type mice, 20 biological replicates, one technical replicate, Supplementary Figure 4C) with the Hoang et al. dataset. Hoang et al., generated two replicates of LD controls and for NMDA controls, respectively, which, since not significantly different, were averaged resulting in 4 technical replicates. Linear regression analysis showed a strong correlation (*R*^2^ = 0.99) of both datasets (Supplementary Figure 4D).

### Most Müller Glia miRNAs Decline After Light Damage

**Figure 3:**
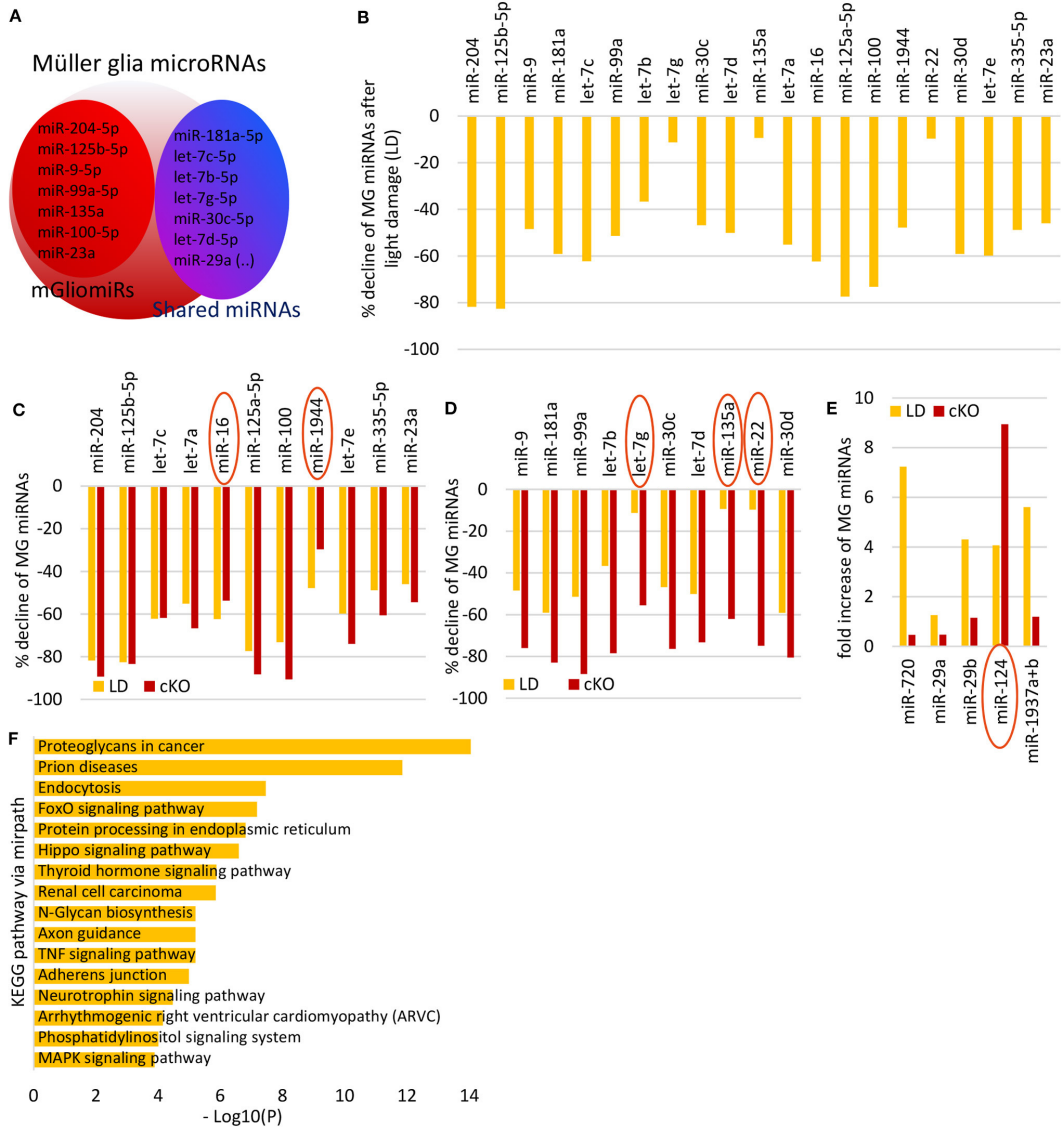
Müller glia miRNA expression after light damage. **(A)** Schematic of miRNA highly expressed in MG i.e., mGliomiRs and shared miRs (modified after Wohl and Reh, 2016). **(B)** Percent decline of the top 21 highly expressed MG miRNAs after 7 days light damage compared to normal MG (cutoff 3,000 counts). **(C)** miRNAs with similar declined levels after light damage as after Dicer deletion (in percent). **(D)** miRNAs with less declined levels after light damage as after Dicer deletion (in percent). **(E)** Fold increase of 5 miRNAs that increased after 7 days light damage (cutoff 5,000 counts) compared to controls. **(F)** DIANA-mirPath v.3 analysis of the 21 MG miRNAs that declined after light damage.

miRNA have been profiled for whole normal retina and after light damage (Karali et al., 2007, 2011; Hackler et al., 2010; Chu-Tan et al., 2020), but not in MG after light damage. In order to profile the miRNAs of reactive MG, we used the MG reporter mice, FACS-purified the MG from undamaged and damaged retinas (7 days after light damage) and performed Nanostring miRNA profiling. Six hundred miRNAs were quantified by solution hybridization using a NanoString nCounter assay. An advantage of this approach is that the NanoString assay does not require amplification, which might introduce bias and requires relatively small amounts of RNA (200 ng), which allows the analysis of small cell populations (Geiss et al., 2008). We focused on the miRNAs that previously were identified to be highly expressed in MG mGliomiRs (<20% expression in neurons) and shared miRs [similar expression levels in neurons (Wohl and Reh, 2016), Figure 3A]. We found that the vast majority of MG miRNAs declined after light damage (Figure 3B, Supplementary Table 4). This result resembled the miRNA changes we previously observed 1 month after Dicer deletion (Wohl et al., 2017). We compared the expression levels to those we obtained after Dicer deletion and found 11 miRNAs similarly reduced, including miR-204, miR-125-5p, and three let-7 family members. Interestingly, miR-16 and miR-1944 expression levels in reactive MG after light damage were lower than in the Dicer-CKO MG (Figure 3C). We also plotted the miRNAs that were less reduced after light damage. These miRNAs included miR-9, miR-181a, and three other let-7 family members (Figure 3D). let-7g, miR-135a, and miR-22 displayed only a reduction of about 10% compared to undamaged controls. Interestingly, from all miRNAs analyzed, only five increased after light damage, i.e., miR-720, miR-29a and b, miR-124-3p, and miR-1937a+b (Figure 3E, Supplementary Table 5). We next used DIANA-mirPath v.3 to see which pathways involve these MG miRNAs. Using the KEGG (Kyoto Encyclopedia of Genes and Genomes) database, we found that most of the MG miRNAs regulate proteoglycans in cancer, prion diseases, FoxO signaling pathway etc. (Figure 3F). Proteoglycans are found in the extracellular matrix (ECM) and plasma membrane of cells and are probably relevant for cell migration. Prions are proteins that trigger abnormal protein folding in the brain leading to neurological disorders. FoxO are a subgroup of forkhead family transcription factors and are involved in cell processes including cell death, DNA repair, cell cycle arrest (Carter and Brunet, 2007).

miRNAs are predominantly found in the cytoplasm of a cell where they act as repressors, being bound to Ago2. However, they can also be found in multivesicular bodies or cell organelles including the nucleus (Leung, 2015). This means a miRNA identified in a cell does not necessarily regulate gene expression. We therefore used Ago HITS-CLIP data of total retina from a recently published study from the Natoli lab (Chu-Tan et al., 2020), to assess if any of the MG miRNAs are bound to Ago2 (Supplementary Figure 5A). Although this dataset is from whole retina and not cell type specific, our previous results comparing MG miRNA expression with retinal neuronal miRNA expression allows us to tentatively assign the HITS-CLIP results for some of the MG-specific miRNAs (mGliomiRs). All MG miRNAs including the mGliomiRs (e.g., miR-204-5p, miR-125b-5p, and miR-9 etc.) and shared miRs (e.g., miR-181a, let-7 family etc.) were bound to Ago2, except miR-1944 (Supplementary Figure 5B). For the upregulated candidates after light damage, we found miR-29a-5p, miR-29b-3p, and miR-124-3p bound to Ago2. Since the data is whole retina, we also plotted the photoreceptor specific miRNAs (miR183/182/96 cluster) as reference, since the vast majority of cells in the whole retina are photoreceptors (Supplementary Figure 5B, Supplementary Table 6).

Taken together, we found that almost all highly expressed MG miRNAs declined 7 days after light damage, a profile that resembled the Dicer-cKO MG 1 month after deletion. We next analyzed the gene expression patterns of reactive MG after light damage and compared them to that found in Dicer-depleted MG.

### Gene Expression Analysis of Reactive Müller Glia After Light Damage and Potential miRNA Regulators

**Figure 4:**
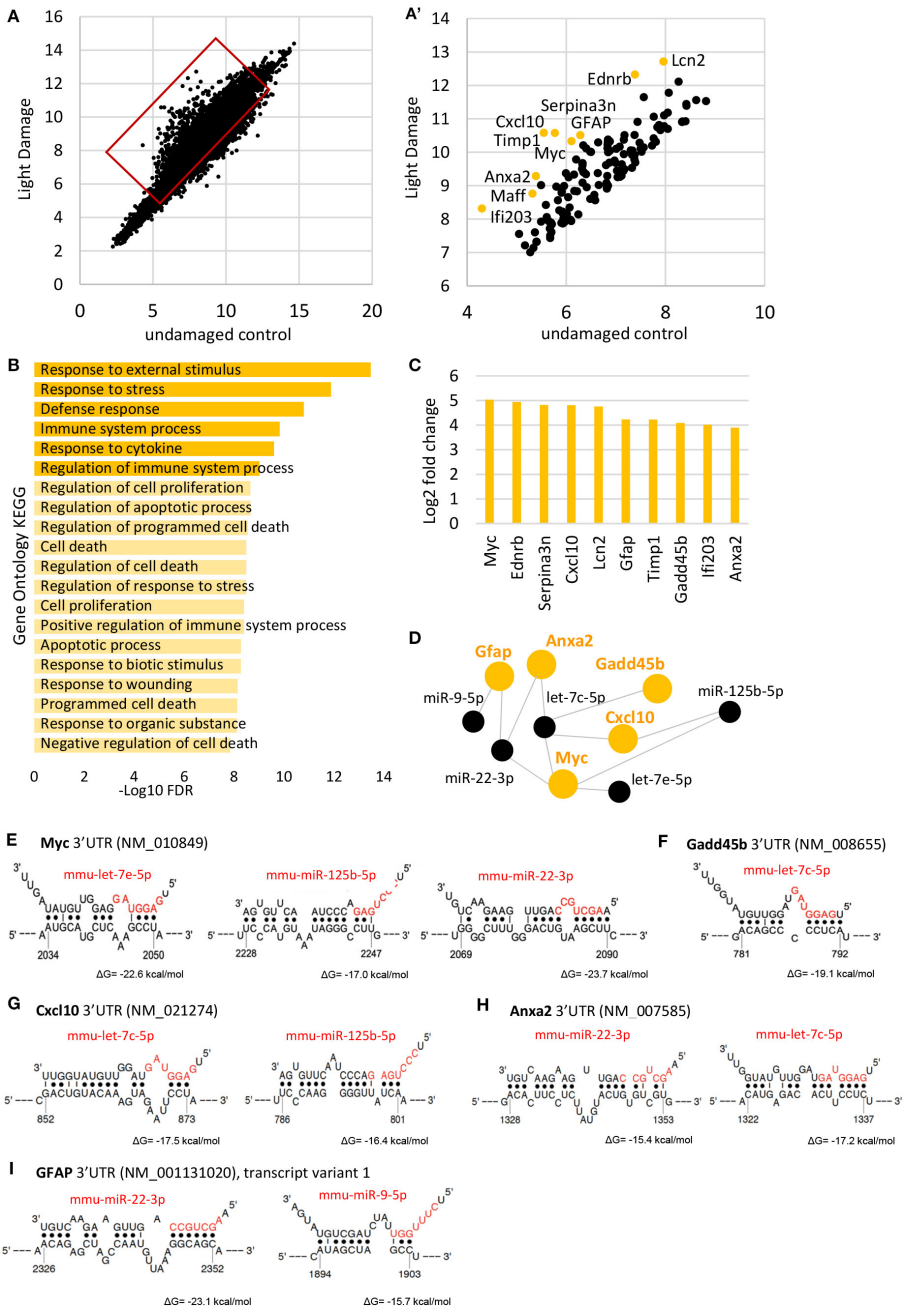
Müller glia gene expression after light damage. **(A)** Scatterplot of genes expressed in undamaged controls and 7 days after light damage (LD) from microarray (log_2_ of relative expression). **(A′)** Scatter plot of the top 100 highly expressed genes after light damage. **(B)** Gene ontology of the top 100 genes upregulated after light damage using ShinyGO v0.61. **(C)** Expression levels of the top 10 genes upregulated after light damage compared to undamaged controls (five and six biological replicates, respectively, one technical replicate per condition). **(D)** DIANA-TarBase v.8 analysis of the top genes upregulated after light damage and MG miRNAs that declined and are likely to target these genes based on prediction and experimental confirmation. **(E–G)** Binding sites of identified miRNAs from D (DIANA-TarBase) in the 3′UTRs of *Myc* **(E)**, *Gadd45b* **(F)**, *Cxcl10* **(G)**, *Anxa2* **(H)**, and *Gfap* **(I)** using STarMirDB. The seed sequences of the particular miRNA are shown in red. Dots represent complimentary base pairing. ΔG indicates the structural accessibility at the target site, low values (e.g., −17 kcal/mol) indicate high accessibility.

For gene expression analysis, we used microarray analysis and plotted the data (log_2_ of relative expression) from light damaged retinas against undamaged controls (Figure 4A). We focused on the top 100 genes upregulated after light damage and carried out gene ontology analysis using ShinyGO (http://bioinformatics.sdstate.edu/go/, Figure 4A′). We found that these genes are involved in processes of stress and defense, cell death/apoptosis, and cell proliferation (Figure 4B). We identified the top 10 highly upregulated genes in the MG 7 days after light damage (at least 3.9-fold increase compared to normal MG expression) which were: *Myc*, (Myelocytomatosis oncogene, alternative symbol c-myc), *Ednrb* (Endothelin receptor type B) *Serpina3n* (Serine (or cysteine) peptidase inhibitor, clade A, member 3N), *Cxcl10* (Chemokine ligand 10), *Lcn2* (Lipocalin 2), *Gfap, Timp1* (*Tissue inhibitor of metalloproteinases), Gadd45b* (Growth arrest and DNA damage inducible beta), *Ifi203* (interferon activated gene 203), and *Anxa2* (Annexin 2) (Figure 4C, Supplementary Table 7). From these genes, *Gfap* has been reported in a variety of studies and is known as a gene for reactive glia. However, less is known about the other genes. So far, *Cxcl10, Myc, Timp1, Serpina3n*, and *Ednrb* have been reported to be upregulated in retinas after light damage (Rattner and Nathans, 2005; Rutar et al., 2015; Mansouri et al., 2020) or other injury/diseases models such as retinal detachment (Rattner and Nathans, 2005), glaucoma (Naskar and Thanos, 2006), or retinal ischemia-reperfusion (Abcouwer et al., 2013). *Lcn2* encoding for lipocalin 2 [also known as NGAL or oncogene 24-3 (Abcouwer et al., 2013)], which was also found to be upregulated in reactive astrocytes (Zamanian et al., 2012) and was reported for whole retina after light damage (Chen et al., 2004); Most of these studies analyzed whole retina, and a MG-specific expression was only confirmed for *Cxcl10* (Rutar et al., 2015), *Ednrb* and *Serpina3n* (Rattner and Nathans, 2005) after light damage. Annexin 2 was shown to be expressed in normal MG (Grosche et al., 2016), but not for reactive glia. We used again the dataset provided by Hoang et al., in order to see if these genes were differentially expressed 36 h after light damage (Hoang et al., 2020). We found all genes increased 36 h post light damage (fold change compared to controls, Supplementary Figure 4E). Please note that the time point of analysis was very early after injury (36 h vs. 7days) and the injury paradigms were different from ours (3,000 lux, 4 h duration vs. 10,000 lux, 8 h duration).

Next, we used DIANA-TarBase to identify the MG miRNAs that might regulate these genes (Karagkouni et al., 2018). DIANA-TarBase is a reference database devoted to the indexing of experimentally supported microRNA (miRNA) targets. It integrates information on cell-type specific miRNA–gene regulation, while thousands of miRNA-binding locations are reported. We analyzed the 21 MG miRNAs that decreased, and the top 10 genes upregulated after light damage. Using this tool, we found 5 of the 10 genes (*Gfap, Anxa2, Cxcl10, Myc*, and *Gadd45b*) are regulated by the miRNAs miR-9-5p, miR-125b-5p, let-7c-5p, and let-7e-5p Figure 4D). In order to identify the binding sites of the miRNAs in the mRNA molecule STarMirDB was used, a database that provides information (sequence, thermodynamic and target structural features) of seed and seedless sites in 3′UTR, CDS and 5′UTR, identified from CLIP (cross-linking immunoprecipitation) data. We found binding sites in the 3′UTR of the particular mRNA molecule for all DIANA-TarBase predicted miRNAs, with high structural accessibility at the target site (ΔG ~ −17 kcal/mol, Figures 4E–I).

The myc-let-7 interactions has been reported for different tissue and species and indicates a conserved mechanism (Chang et al., 2009; Kim et al., 2009; Ramachandran et al., 2010; Buechner et al., 2011; Leppert et al., 2011; Wong et al., 2011; Gunzburg et al., 2015; Maldotti et al., 2016; Balzeau et al., 2017). Interestingly, we did not find any of these mRNAs bound in the whole retina Ago HITS-CLIP data set, except *Gfap*. This could imply an indirect regulation via other genes. It is also possible that these genes were not detectable due to low MG input (since MG comprise only 2% of the retina). It was shown before that strong signals in MG-enriched samples might not be detected in whole retina samples (Diaz Quiroz et al., 2014). However, *Gfap* together with other glial markers such as *Glul* (glutamine synthetase), *Scl1a3* (glutamate aspartate transporter GLAST), *Sox2, Sox9, Vim* (Vimentin), and *Aqp4* (Aquaporin4) were bound to Ago2 in RISC, suggesting that the transcripts of these genes are regulated by miRNAs in retinal cells (Supplementary Figure 5C).

**Figure 5:**
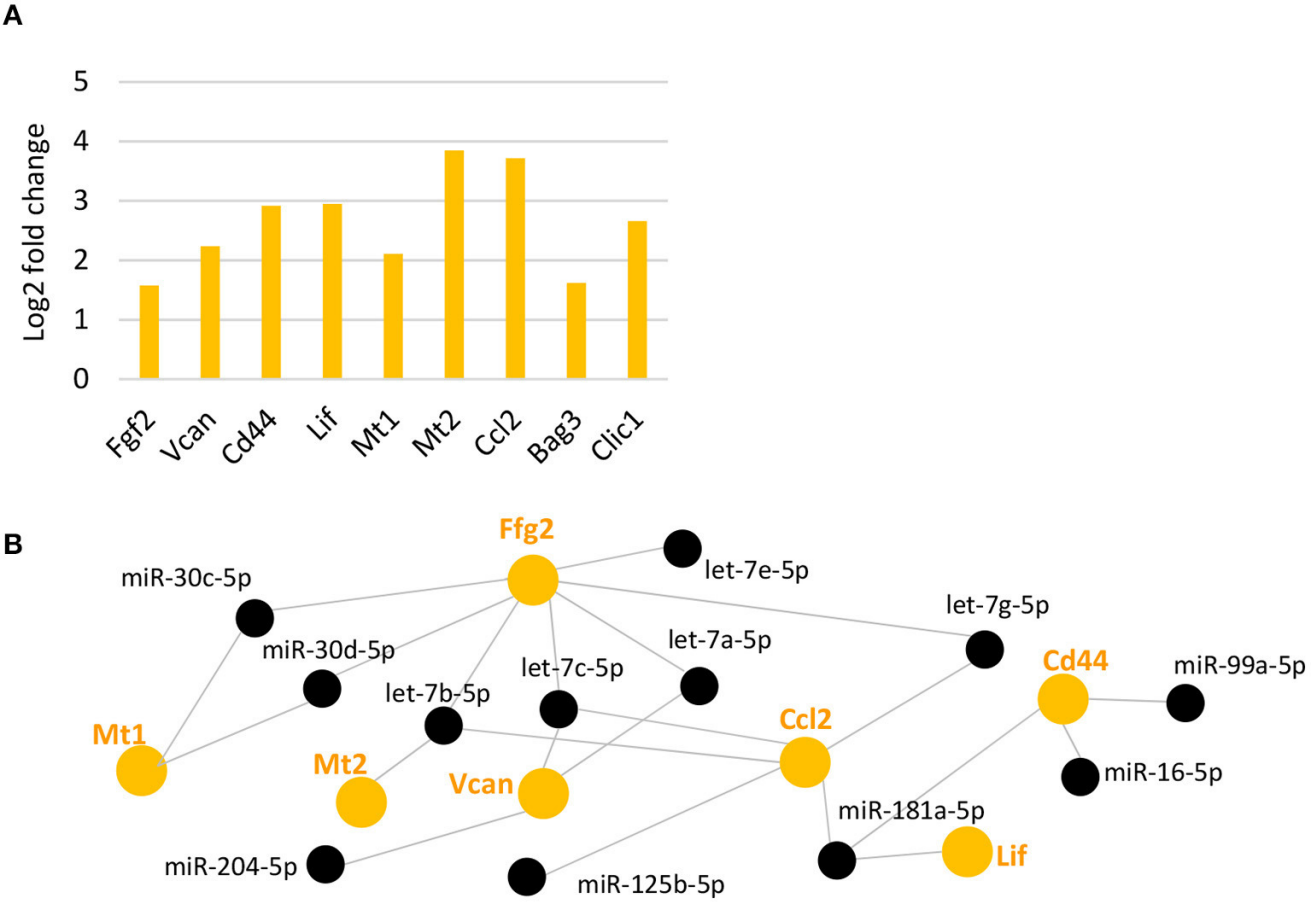
Upregulated reported genes after light damage and their potential miRNA regulators. **(A)** Expression levels (fold change of log_2_ relative expression) of reported genes upregulated after light damage compared to undamaged controls (five and six biological replicates, respectively, one technical replicate per condition). **(B)**: DIANA-TarBase analysis of genes in A and MG miRNAs that declined and are likely to target these genes based on prediction and experimental confirmation. FDR, false discovery rate.

We next analyzed genes that have been reported in previous studies to be upregulated after light damage in MG (Grosche et al., 1995; Hartig et al., 1995; Ueki et al., 2008; Bringmann et al., 2009) or whole retina (Chen et al., 2004) (see complete list in Supplementary Table 8). From this list of genes, we found 10 genes with an at least 3.9-fold increase in gene expression 7 days after light damage (Figure 5A). These genes included: *Fgf2*, encoding for the fibroblast growth factor 2, which is known to be expressed in MG after damage (Guillonneau et al., 1998); *Vcan* and *Cd44*, encoding for the extracellular matrix protein versican and the cell surface receptor CD44 (CD44 antigen) respectively [see review Bringmann et al., 2009)]; *Lif* , encoding for leukemia inhibitory factor that actives the STAT (signal transducer and activator of transcription) pathway (Ueki et al., 2008); *Mt1* and *Mt2* encoding for the proteins metallothionein 1 and 2, found in whole retina after light damage (Chen et al., 2004); *Ccl2* encoding the chemokine (C-C motif) ligand 2 (Abcouwer et al., 2013; Chu-Tan et al., 2018; Mansouri et al., 2020); *Bag3*, encoding for BCL2-associated athanogene 3, and *Clic1*, encoding for the chloride channel protein chloride intracellular channel 1. Both, *Bag3* and *Clic1* were reported for light damaged retinas (Chen et al., 2004). Although the light damage paradigms and/or time points of analysis from the other studies were different to ours, we found the same genes upregulated. We next used DIANA-TarBase to see if MG miRNAs regulate these genes. We found that *Fgf2, Vcan, Cd44, Lif* , *Mt1*, and *Mt2*, as well as *Ccl2* are regulated by MG miRNAs and predominantly by members of the let-7 family (Figure 5B). Interestingly, *Mt2, Lcn2, Ccl2, Bag3*, and *Clic1* were also found to be bound to Ago2 in RISC (Supplementary Figure 5D).

Taken together, light damage causes a decline in the vast majority of MG miRNAs, which might in turn be responsible for some of the increases in specific genes that occur in MG in response to the injury. Among the top genes upregulated in reactive MG, most were bound to Ago2 in the HITS-CLIP data and many are potential targets of mGliomiRs.

### Genes Similarly Upregulated in Reactive and Dicer-Depleted Müller Glia and Their Potential miRNA Regulators

**Figure 6:**
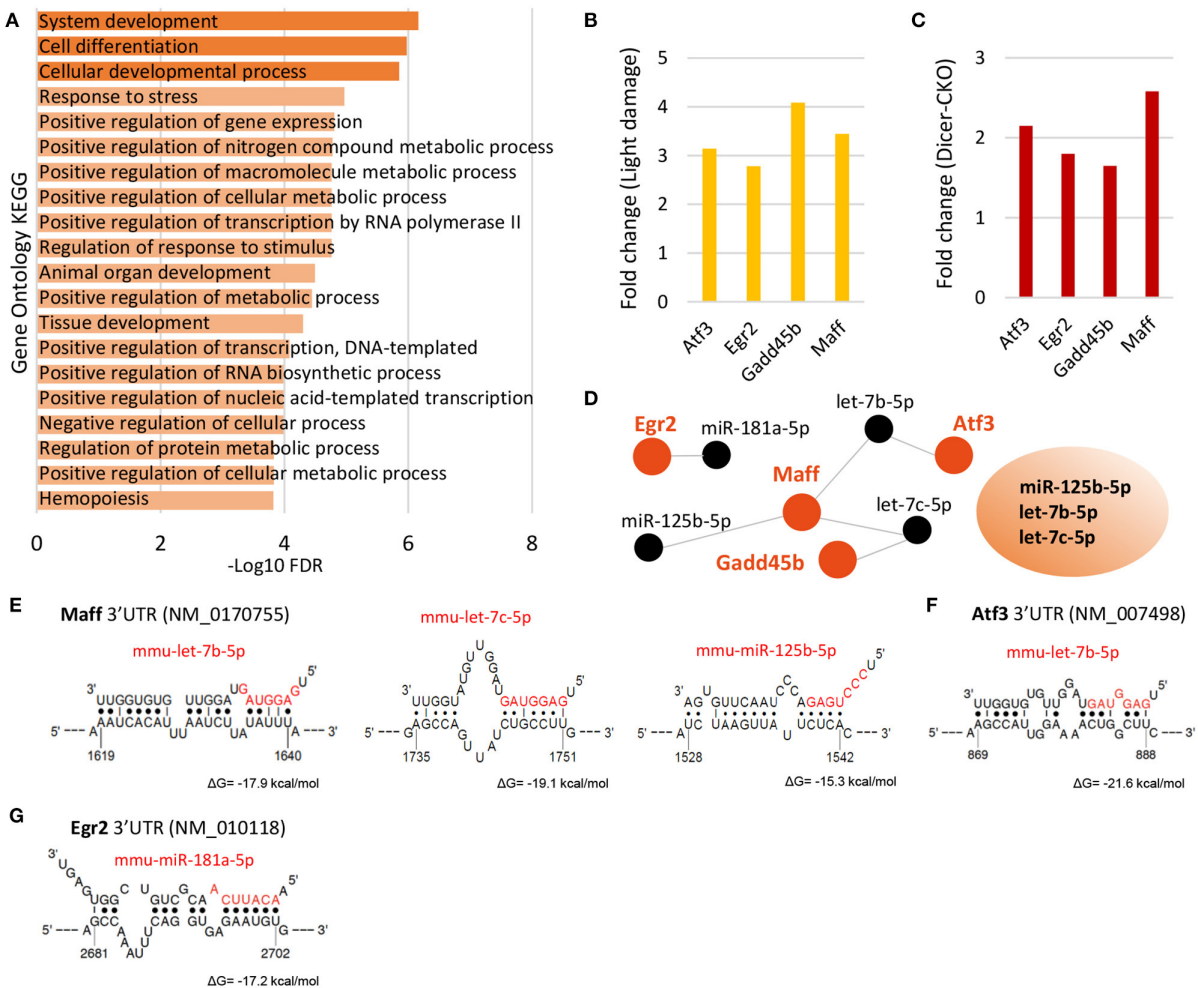
Upregulated genes after light damage and after Dicer deletion and their potential miRNA regulators. **(A)** Gene ontology of the top 20 genes upregulated the most after Dicer deletion and light damage (LD), FDR: false discovery rate. **(B,C)** Top four genes upregulated after light damage (**B**, fold change of log_2_ relative expression of light damage vs. controls, five and six biological replicates, respectively, one technical replicate per condition) or Dicer deletion (**C**, fold change of log_2_ counts per million of wild type or Dicer-deleted MG, 20 and 14 biological replicates, respectively, 1 technical replicate per condition). **(D)** DIANA-TarBase predicted miRNAs regulating the top four genes (*Atf3, Egr2, Gadd45b*, and *Maff* ) upregulated in the Dicer-cKO and after light damage and miRNAs found to be regulating the top genes after LD and the top 4 genes in the light damage/Dicer-cKO dataset i.e., miR-125b-5p, let-7c-5p, and let-7b-5p. **(E–H)** Binding sites of identified miRNAs from D (DIANA-TarBase) in the 3′UTRs of Maff **(E)**, Atf3 **(F)**, and Egr2 **(G)** using STarMirDB. The seed sequences of the particular miRNA are shown in red. Dots represent complimentary base pairing. ΔG indicates the structural accessibility at the target site.

In our previous analysis of the changes in the transcriptome of MG after Dicer-cKO, we identified Bcan as the most highly upregulated gene. Given the similarities in the miRNA reduction in the Dicer-cKO and light damaged MG, we compared the changes in gene expression in these experimental paradigms. We compared all genes that had a minimum of log_2_ 5.0 in the light damage dataset and a minimum of log_2_ CPM 4.0 in the RNA-Seq dataset. Twenty genes had an at least a 1.3 fold change after light damage or at least a 0.7 fold increase in the Dicer-cKO (Supplementary Table 9). We did gene ontology with these 20 genes and found that they are involved in response to developmental, metabolic and stress processes (Figure 6A). Four genes were found in both data sets, which had an at least a 2.7 and 1.4 fold increase after light damage or Dicer-cKO, respectively, which were the stress induced transcription factor *Atf3* (activating transcription factor 3 or also known as cyclic AMP-dependent transcription factor 3), *Egr2* (early growth response 2), *Gadd45b* (growth arrest and DNA damage inducible beta), and *Maff* (MAF BZIP Transcription Factor F) (Figures 6B,C). *Atf3* and *Gadd45*b were also bound to Ago2 (Supplementary Figure 5E).

In order to identify the miRNAs that potentially regulate the genes, we used DIANA-TarBase and found the miRNAs miR-125b-5p, miR-181-5p, let-7b, and let-7c are potential regulators of these genes (Figure 6D). We then compared the miRNA:mRNA network with that after light damage (Figure 4D) and found that three microRNAs were present in both networks. These miRNAs were: miR-125-5p and let-7b and let-7c. This suggests that this miRNA trio might play an important role in the changes in gene expression in MG that accompany gliosis. We used STarMirDB to identify the binding sites of the miRNAs in the mRNA molecule and found binding sites in the 3′UTR of the particular mRNA molecule for all DIANA-TarBase predicted miRNAs, with high structural accessibility at the target site (ΔG ~ −17 kcal/mol, Figures 4F, 6E–G).

### Dicer and the RISC Appear Not to Be Influenced by Light Damage or Dicer Deletion

**Figure 7:**
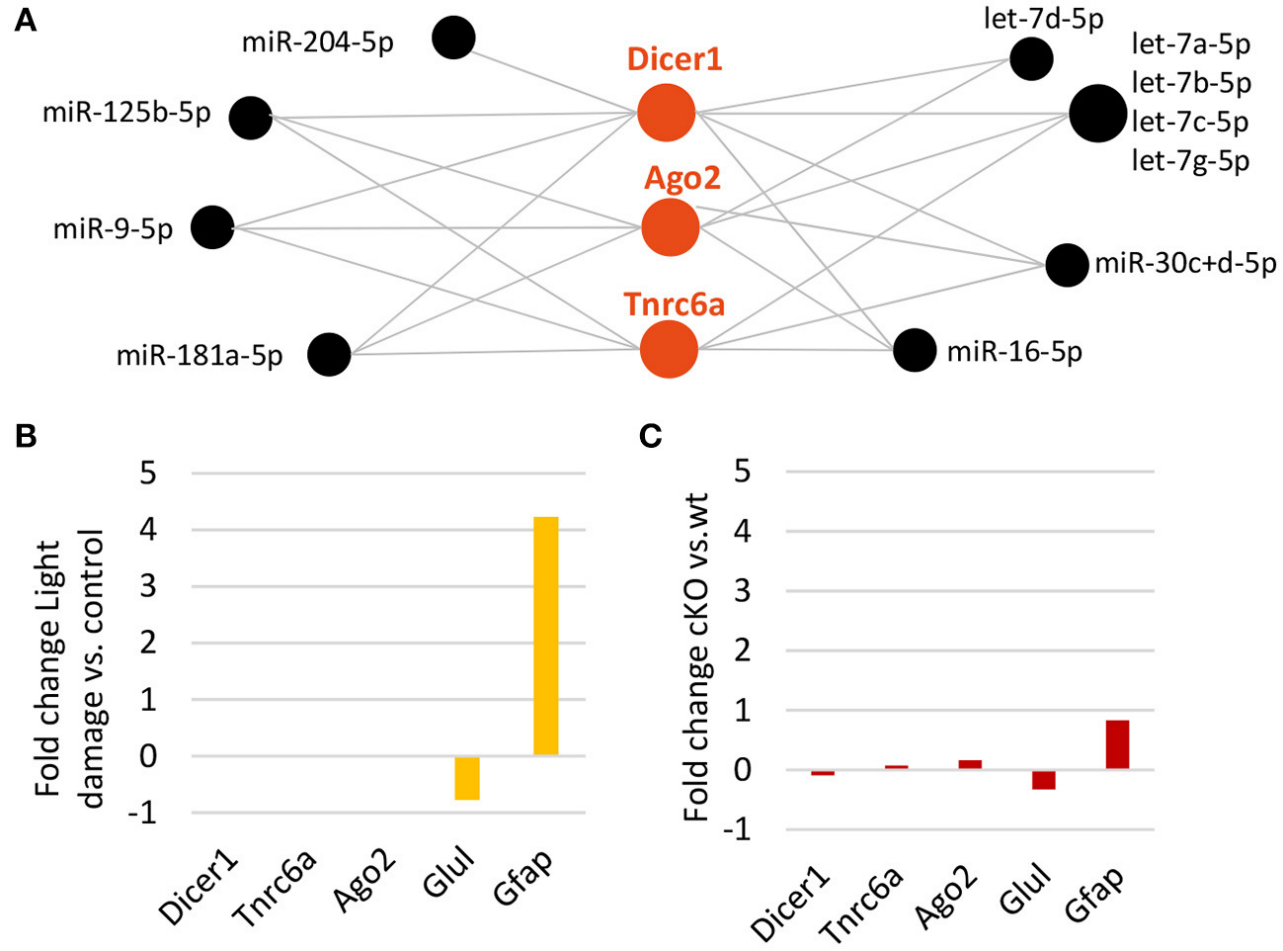
Decline in Müller glia miRNAs does not affect Dicer or RISC. **(A)** DIANA-TarBase predicted/confirmed miRNAs regulating Dicer1, or members of the RNA-induced silencing complex (RISC) such as Argounate2 and or after Dicer deletion. **(B)** Expression levels (fold change of log_2_ relative expression) of *Dicer1*, RISC genes, *Glul* and *Gfap* MG genes (reference) after light damage vs. controls (five and six biological replicates, respectively, one technical replicate per condition). **(C)** Expression levels (fold change of log_2_ counts per million) from RNA-seq of *Dicer1, Tnrc6a, Ago2, Glul*, and *Gfap* after Dicer deletion vs. wild type (14 and 20 biological replicates respectively, one technical replicate per condition).

It is known that Dicer is regulated by miRNAs, in particular by let-7 (Tokumaru et al., 2008). Since we found a significant reduction in MG miRNAs including the let-7 family, we addressed the question whether or not Dicer or members of the RISC complex such as Ago2 or GW182 (encoded by *Tnrc6a*) are affected. DIANA-TarBase revealed eleven miRNAs, which are highly expressed in the MG, have been shown to target all three genes. Among them are the mGliomiRs miR-204, miR-125b, miR-9 as well as five let-7 family members (Figure 7A). However, when we compared the expression levels after light damage with undamaged controls, no difference for *Dicer1* or *Tnrc6a* expression levels was found (Figure 7B, Ago2 was not found among the genes on the microarray). In addition, none of the three genes were differently expressed after Dicer deletion (Figure 7C, Glutamine synthetase and GFAP levels plotted as reference). Ago HITS-CLIP showed that only Ago2 was found to be bound (Supplementary Figure 5F). This implies that the loss of miRNAs, at least at these time points of analysis, had no effect on Dicer expression or on the expression of two members of the RISC complex.

## Discussion

We previously profiled the miRNAs highly expressed in MG (Wohl and Reh, 2016) and found that they are required for proper MG function, which in turn is required for proper retinal architecture and retinal health (Wohl et al., 2017). In this study, we went on to profile miRNAs in reactive MG after light damage to the rod photoreceptors. We compared the miRNA and mRNA expression pattern in MG from light damaged (7 days) retinas with those from the Dicer-cKO mice (1 month) and found that the vast majority of miRNAs highly expressed in adult MG declined, while only a few increased. We found that genes upregulated in MG after light damage were the same as reported in other damage studies on whole retina and identified their potential miRNA regulators using DIANA-TarBase. DIANA-TarBase is a tool that combines computational prediction and a database of experimentally validated miRNA:mRNA interactions from over 500 tissues, mostly done by HITS-CLIP (Karagkouni et al., 2018). The comparison with the Dicer-cKO MG revealed striking similarities with regard to miRNAs levels, which implied similar genes to be affected in both conditions. We found four genes*, Maff, Egr2, Atf3*, and *Gadd45b* present in both datasets and three miRNAs, which might be potential key regulators in MG dysfunction, namely miR-125b-5p, let-7b-5p, and let-7c-5p.

### Structural, Cellular, and Molecular Similarities and Differences in Retinas After Light Damage and After Müller Glia-Specific Dicer Deletion

We previously reported that deletion of Dicer1 specifically in MG (Dicer-cKO) causes a rod photoreceptor degeneration and retinal disorganization that resembles end stage retinitis pigmentosa (Wohl et al., 2017). At later stages after Dicer deletion, we found a substantial loss of photoreceptor cells that started in the central retina and spreads toward the periphery over time. The retinal remodeling that occurs in later stages of retinitis pigmentosa and other retinal degenerations is dominated by MG hypertrophy that fills the gaps in the tissue as neurons degenerate (Jones et al., 2003; Marc and Jones, 2003; Jones and Marc, 2005; Marc et al., 2007). How reactive gliosis and this glial-driven remodeling are regulated is, however, still unknown. When we compared light damaged retinas with Dicer-cKO^MG^ retinas at later time points (Wohl et al., 2017), the structural and cellular similarities we found were significant thinning of the center of the retina due to photoreceptor loss, MG migration toward the ONL and loss of glutamine synthetase expression as a result of the loss of glutamatergic neurons (Bringmann et al., 2006, 2009). The comparison of miRNA and transcriptomic changes in the MG revealed that under both conditions, all highly expressed MG miRNAs (mGliomiRs and shared miRs) were substantially reduced. Thus, the miRNA profile of reactive MG was similar to that of Dicer depleted MG. However, despite the overall decline, the absolute levels of some miRNAs were different for reactive MG and Dicer-cKO MG. This suggests that specific levels of miRNAs are responsible for particular gene targeting. Since all MG miRNAs were also found to be bound to Ago2, they are indeed regulatory miRNAs in retinal cells (Chi et al., 2009; Chu-Tan et al., 2020). This suggests that MG miRNAs play a role in reactive gliosis.

We also found 5 miRNAs upregulated 7 days after light damage, i.e., miR-720, miR-29a, miR-29b, and miR-1937a/b. Note, miR-720 is no longer considered as a miRNA, but is now known to be a fragment of a tRNA (Schopman et al., 2010). However, it has been reported as a miRNA involved in cell proliferation, differentiation, and migration (Hara et al., 2013; Torres-Martin et al., 2013; Li et al., 2014; Tang et al., 2015). One miRNA upregulated after light damage also showed an increase after Dicer deletion: miR-124. Although there are miRNAs that do not require Dicer for their processing (Cheloufi et al., 2010), miR-124 appears not to be among them (Huyghe et al., 2015). Therefore, this increase is probably due to neuron-glia communication via exosomes, with miR-124 as the predominant miRNA (Morel et al., 2013; Men et al., 2019; Wooff et al., 2020). Increased neuron-glial communication via exosomes has been reported after light damage with miR-124 as the most abundant cargo (Wooff et al., 2020).

### Maff, Atf3, Egr2, and Gadd45b and Their Potential Role in Müller Glia

To better understand the common factors that might be driving gliosis, we compared gene expression of reactive MG after light damage with MG from Dicer-cKO mice. We found four genes that had at least 2.8 fold increase after light damage and 1.6 fold increase in Dicer-deleted MG which were *Atf3, Egr2, Maff*, and *Gadd45b*. Not much is known about the function of these genes in MG; however, recently, *Atf3, Egr2*, and *Maff* have been identified as murine MG-expressed transcription factors that are involved in TNFα signaling and were also found to be upregulated 36 h after light damage in GlastCre-GFP+ FACS-purified MG (Hoang et al., 2020). TNFα, also known as TNF, is the master pro-inflammatory cytokine, and has been shown to be involved and modulate multiple signaling pathways with wide-ranging downstream effects. It is a multifunctional molecule involved in the regulation of a wide spectrum of biological processes including cell proliferation, differentiation, and apoptosis. Reactive MG release TNFα [see reviews (Bringmann et al., 2006, 2009)] however, we did not find TNFα upregulated in the reactive MG 7 days after light damage, nor after Dicer deletion.

*Maff* is a member of the small Maf family proteins that can activate or repress transcription, but this transcription factor has not been reported in connection with glia or gliosis. It has been reported to be associated with stress response, which links it to disease pathologies including neurological diseases. These neurological disorders and diseases include schizophrenia (SCZ), bipolar disorder (BD), and major depressive disorder (MDD) (Lanz et al., 2019) as well as Parkinson's disease and Alzheimer's disease (Wang et al., 2017). Interestingly, *Maff* can bind to Nuclear factor erythroid 2-related factor 2 (*Nrf2*, encoded by the *Nfe2l2* gene), a transcription factor that regulates the expression of antioxidant proteins. *Nrf2* has been reported in a variety of studies about retinal injury such as hypoxia, ischemia and light damage, and an activation of NRF2 by different compounds/factors has been shown to reduce the effect of MG gliosis (Tan et al., 2015; Deliyanti et al., 2016; Inoue et al., 2017). We did not find differentially expressed *Nfe2l2* (whose levels are also rather low in MG) after light damage or Dicer deletion. Nevertheless, *Maff* and its role in regulating stress response by binding to *Nfe2l2* could be relevant for regulating gliosis.

*Atf3* is a member of the activating transcription factor/cAMP responsive element binding (CREB) protein family of transcription factors, which share the basic region-leucine zipper (bZip) DNA binding motif. The level of *Atf3* mRNA is low or undetectable in normal mouse tissue and most cell lines but increases significantly after stimulation. *Atf3* is induced by a variety of stress signals including ER stress, stresses that induce integrated stress response, and other stress signals. One striking feature of *Atf3* induction is that it is neither tissue-specific nor stimulus-specific; it can be induced by a broad spectrum of stimuli and can be induced in various tissues or cell types [see (Hai et al., 2011)]. *Atf3* plays a role in metabolic regulation, immune response, and ontogenesis. *Atf3* increases various downstream targets such as *Ccl2* (which is also upregulated after light damage Figure 4E), induces cell proliferation but also apoptosis (Ku and Cheng, 2020). In the retina, it has been described in fish regeneration (Saul et al., 2010), as a neuroprotective factor after optic nerve crush in murine neurons (Kole et al., 2020) and as a cyto-protective factor in astrocytes after oxidative stress induction (Kim et al., 2010). For MG, *Atf3* is suggested to be regulated by tumor suppressor protein p53 (Ueki et al., 2012). *Atf3* is also reported to be induced by ciliary neurotrophic factor (CNTF), a member of the interleukin-6 cytokine family that is suggested as an inducer of gliosis (Xue et al., 2011). In the same study, *Atf3* was found to be expressed together with *Egr2* (also termed *Krox20*, which plays an important role in neuronal development). Interestingly, both proteins, Atf3 and Egr2, have also been reported in a study about MG that underwent cyclic mechanical stretching (Wang et al., 2013). The retina in diseases such as myopia or proliferative vitreoretinopathy is often subjected to mechanical forces which lead to tissue deformation and consequently MG stretching. It is interesting that we find two of these genes after Dicer deletion in MG, since the retinas of these mice had puffy/stretched areas. However, we interpreted these areas as a consequence of changes in MG gene expression due the miRNA decline (Wohl et al., 2017). Moreover, it is also very intriguing that these genes are found after light damage, since light damage leads to a significant retinal thinning and causes the opposite of tissue stretching. *Egr2* has also been reported to be upregulated in astrocytes after increased intraocular pressure. This model induces a mechanical stress response that could also be considered as a different kind of cellular stretching (Yang et al., 2004) and implies a common gene for retinal MG and optic nerve head astrocytes. *Gadd45b* (Growth Arrest and DNA-Damage-inducibleprotein45 beta, also known as MyD118) belongs to the highly homologous Gadd45 family of proteins. These proteins are found in the nucleus and are known to act as stress-response genes (Liebermann and Hoffman, 2007). They function in DNA repair, apoptosis, cell survival, growth arrest, and probably DNA demethylation (Barreto et al., 2007). DNA demethylation typically activates gene expression, while DNA methylation is one of the various epigenetic mechanisms for silencing gene expression (Reik, 2007). Altered DNA methylation also occurs in pathological processes, such as silencing of tumor repressor genes in cancer cells. *Gadd45b* was shown to be induced by neuronal activity, which promoted epigenetic DNA demethylation and adult neurogenesis (Ma et al., 2009). However, whether *Gadd45b* has a similar function in glia, in particular in MG, is not known yet.

### miR-125b-5p, let-7b-5p and let-7c-5p as Potential Key Regulators in Müller Glia

We found three miRNAs, miR-125b, let-7c, and let-7b, as potential regulators of genes after light damage and in the dataset of light damage/ Dicer-cKO using DIANA-TarBase. This implies that these miRNAs might play an important role for glial function. To our knowledge, none of them has been reported yet in the context of reactive MG. let-7 and miR-125 are known to play an important role during retinogenesis (transition from early to late retinal progenitor cells) and this is a conserved pathway across vertebrates (La Torre et al., 2013).

However, in other regions of the CNS, i.e., the brain or spinal cord, miR-125b has been reported to play a role in glial scar formation. A study analyzing the environment after spinal cord injury by cross-species comparison between salamander and rat revealed that precise levels of miR-125b can recreate a permissive environment for axon regeneration in rat by targeting the semaphorin gene *Sema4* (Diaz Quiroz et al., 2014). It was shown that in normal spinal cords, the radial glia cells in salamander express very high levels of miR-125b. Rat astrocytes, however, have low levels. After spinal cord transection, the levels in the salamander spinal cords drastically decreased by 40%, while the levels in rat astrocytes did not change. When miR-125 was overexpressed in rats using mimics, a reduction of the glial scar (downregulation of genes involved in glial scar formation such as *Gfap, Cspg4* and *Col6A1*) and an increase of the number and length of axons projecting into the scar were observed (Diaz Quiroz et al., 2014). Although the authors showed that this mechanism is conserved across vertebrates, it appears that this mechanism is astrocyte specific. In our dataset, *Sema4, CSPG4*, and *Col6A1* were expressed at very low levels in MG and no change in gene expression was found either after light damage or after the Dicer deletion. This implies that astrogliosis in spinal cord is different from MG gliosis which might be due to the fact that astrocyte miRNAs (low miR-125b) are different from MG miRNAs (high miR-125b).

Another study about normal human brain astrocytes reported that treatment with interleukin IL-6, which induces cellular stress, leads to upregulation of miR-125b and *Gfap* as well as downregulation of cyclin-dependent kinase Cdkn2a, a negative regulator of cell proliferation (Pogue et al., 2010). This correlation was also observed in advanced Alzheimer's disease (Lukiw, 2007). This might suggest that astrogliosis in the human brain is different than astrogliosis in the rat spinal cord since miR-125 levels are different. *Cdkn2a* levels were low in MG and not changing after light damage or Dicer-deletion further implying that MG gliosis and astrogliosis are different. However, this raises the question which miRNAs are found in astrocytes. Rao et al., reported the miRNAs highly expressed in astrocytes of fetal and adult normal brains namely miR-99a, miR-143, and miR-449 (Rao et al., 2016). Interestingly, miR-99a is also among the highly expressed miRNAs in MG (Wohl and Reh, 2016). They also listed the miRNAs functionally characterized in astrocytes, which included miR-125b and two other miRNAs, which are also found to be expressed in MG, i.e., miR-181a, and miR-100 (Rao et al., 2016). Although levels of miR-125b in astrocytes were reported to be low, Shenoy et al. reported that miR-125b and let-7 are required for astrogliogenesis, meaning the transition from glial progenitor cells into astrocytes (Shenoy et al., 2015). This suggests a role for these miRNAs in glial identity.

The let-7 family members are known to act as tumor suppressors and regulators of developmental processes and that their biogenesis is tightly controlled (Lee and Dutta, 2007; Lee et al., 2011, 2016). Recently, let-7e and let-7i have been described as inhibitors for MG-derived retinal regeneration in Royal College of Surgeon rats (Tao et al., 2016). Let-7 targets Lin-28, a transcription factor, which is essential in fish regeneration (Ramachandran et al., 2010) and Lin-28 itself regulates let-7 expression (feedback loop). Ectopic expression of Lin-28 resulted in decreased accumulation of let-7 miRNAs and promoted MG de-differentiation *in vivo* with subsequent neurogenesis and inhibition of gliogenesis. This confirms the hypothesis of a role in glial identity. However, in our data set, let-7 family members declined but Lin-28 was not found to be increased after let-7 reduction in both of our conditions, light damage and Dicer-cKO.

### Similarities and Differences of Müller Glia Gene Expression After Light Damage in Fish

Fish MG have the ability to de-differentiate into a retinal progenitor that can give rise to all retinal neuron types and regenerate the retina (Hitchcock et al., 2004; Bernardos et al., 2007; Kassen et al., 2007; Fausett et al., 2008; Fischer and Bongini, 2010; Karl and Reh, 2010; Ramachandran et al., 2010; Goldman, 2014; Lenkowski and Raymond, 2014; Wan and Goldman, 2016; Otteson, 2017; Elsaeidi et al., 2018). In experiments with fish conduction light damage, MG undergo complex phenotypic changes, enter the cell cycle only within a day and generate new neurons (Yurco and Cameron, 2005; Vihtelic et al., 2006; Kassen et al., 2007; Thummel et al., 2008). Mammals, however, lack that capacity since proliferative and neurogenic competence are both suppressed by a dedicated gene regulatory network in mouse MG that have been recently revealed by Hoang and colleagues (Hoang et al., 2020). The comparison of differentially expressed genes in fish and mouse MG after damage showed that rapidly induced genes in fish were enriched for ribosome biogenesis, protein folding and VEGF signaling pathway. In mouse, however, they included components of the tumor necrosis factor (TNF), nuclear factor kB (NFkB), mitogen-activated protein kinase (MAPK), and Hippo pathways. Slowly induced mouse genes were enriched for ribosome biogenesis and proteasome, whereas in zebrafish, they were enriched for cell cycle| related functions and DNA replication (Hoang et al., 2020). Genes upregulated in both species include *Gfap* and *Stat3* however, the downstream events, especially of the STAT-pathway, are different which is primarily due to epigenetics (Kassen et al., 2007; Ueki et al., 2008; Hong et al., 2014; Hoang et al., 2020; Jorstad et al., 2020). The possibility that this differential regulation could be partially caused by miRNAs has to our knowledge not been explored yet.

Taken together, here we report the miRNA and mRNA profile found in reactive MG 7 days after light damage. We found that the vast majority of MG miRNAs declined and that this profile resembled the MG profile 1 month after MG-specific Dicer deletion, regardless of the extend of the neuronal loss. By analyzing the genes upregulated after light damage, four genes were found to be upregulated in both data sets, *Atf3, Egr2, Gadd45b*, and *Maff* . These genes have not been well-studied in MG yet. Nevertheless, they are associated with stress responses that appear to occur in damage-induced models (direct neuronal damage), and in models of dysregulated MG, here induced by the loss of Dicer1. This implies there is a common unspecific MG stress response that is regulated by miRNAs, potentially regulated by miR-125b, let-7c, and let-7b. This result opens up the possibility that the MG stress response could be altered by manipulating these miRNAs. Subsequent downstream experiments will reveal whether manipulation of these genes has an impact on gliosis, which would have clinical relevance for various types of retinal diseases, independent of the primary cause.

## Data Availability Statement

mRNA and miRNA datasets for wild type undamaged control MG and light damaged MG are available at GEO (GSE163754).

## Ethics Statement

The animal study was reviewed and approved by Institutional Animal Care and Use Committee (IACUC). Written informed consent was obtained from the owners for the participation of their animals in this study.

## Author Contributions

SW and TR conceived the study. SW, DL, SK, SR, and MA conducted experiments. SW and SR conducted data collection. SW and TR analyzed the data. SW, SK, and DL wrote the manuscript with inputs from co-authors. All authors contributed to the article and approved the submitted version.

## Conflict of Interest

The authors declare that the research was conducted in the absence of any commercial or financial relationships that could be construed as a potential conflict of interest.

## References

[B1] Abcouwer S. F. Lin C. M. Shanmugam S. Muthusamy A. Barber A. J. Antonetti D. A. (2013). Minocycline prevents retinal inflammation and vascular permeability following ischemia-reperfusion injury. J. Neuroinflammation 10:149. 10.1186/1742-2094-10-149 24325836 PMC3866619

[B2] Balzeau J. Menezes M. R. Cao S. Hagan J. P. (2017). The LIN28/let-7 pathway in cancer. Front. Genet 8:31. 10.3389/fgene.2017.00031 28400788 PMC5368188

[B3] Barreto G. Schafer A. Marhold J. Stach D. Swaminathan S. K. Handa V.. (2007). Gadd45a promotes epigenetic gene activation by repair-mediated DNA demethylation. Nature 445, 671–675. 10.1038/nature05515 17268471

[B4] Basak O. Taylor V. (2007). Identification of self-replicating multipotent progenitors in the embryonic nervous system by high Notch activity and Hes5 expression. Eur. J. Neurosci. 25, 1006–1022. 10.1111/j.1460-9568.2007.05370.x 17331197

[B5] Beatty S. Koh H. Phil M. Henson D. Boulton M. (2000). The role of oxidative stress in the pathogenesis of age-related macular degeneration. Surv. Ophthalmol. 45, 115–134. 10.1016/S0039-6257(00)00140-5 11033038

[B6] Bernardos R. L. Barthel L. K. Meyers J. R. Raymond P. A. (2007). Late-stage neuronal progenitors in the retina are radial Müller glia that function as retinal stem cells. J. Neurosci. 27, 7028–7040. 10.1523/JNEUROSCI.1624-07.2007 17596452 PMC6672216

[B7] Bringmann A. Francke M. Pannicke T. Biedermann B. Kodal H. Faude F.. (2000). Role of glial K(+) channels in ontogeny and gliosis: a hypothesis based upon studies on Muller cells. Glia 29, 35–44. 10.1002/(SICI)1098-1136(20000101)29:1&lt;35::AID-GLIA4&gt;3.0.CO;2-A 10594921

[B8] Bringmann A. Iandiev I. Pannicke T. Wurm A. Hollborn M. Wiedemann P.. (2009). Cellular signaling and factors involved in Muller cell gliosis: neuroprotective and detrimental effects. Prog. Retin. Eye Res. 28, 423–451. 10.1016/j.preteyeres.2009.07.001 19660572

[B9] Bringmann A. Pannicke T. Grosche J. Francke M. Wiedemann P. Skatchkov S. N.. (2006). Müller cells in the healthy and diseased retina. Prog. Retin. Eye Res. 25, 397–424. 10.1016/j.preteyeres.2006.05.003 16839797

[B10] Bringmann A. Reichenbach A. (2001). Role of Muller cells in retinal degenerations. Front. Biosci. 6, E72–92. 10.2741/Bringman 11578954

[B11] Buechner J. Tomte E. Haug B. H. Henriksen J. R. Lokke C. Flaegstad T.. (2011). Tumour-suppressor microRNAs let-7 and mir-101 target the proto-oncogene MYCN and inhibit cell proliferation in MYCN-amplified neuroblastoma. Br. J. Cancer 105, 296–303. 10.1038/bjc.2011.220 21654684 PMC3142803

[B12] Burns M. S. Robles M. (1990). Muller cell GFAP expression exhibits gradient from focus of photoreceptor light damage. Curr. Eye Res. 9, 479–486. 10.3109/02713689008999613 2200639

[B13] Carter M. E. Brunet A. (2007). FOXO transcription factors. Curr. Biol. 17, R113–114. 10.1016/j.cub.2007.01.008 17307039

[B14] Chang T. C. Zeitels L. R. Hwang H. W. Chivukula R. R. Wentzel E. A. Dews M.. (2009). Lin-28B transactivation is necessary for Myc-mediated let-7 repression and proliferation. Proc. Natl. Acad. Sci. U.S.A. 106, 3384–3389. 10.1073/pnas.0808300106 19211792 PMC2651245

[B15] Cheloufi S. Dos Santos C. O. Chong M. M. Hannon G. J. (2010). A dicer-independent miRNA biogenesis pathway that requires Ago catalysis. Nature 465, 584–589. 10.1038/nature09092 20424607 PMC2995450

[B16] Chen L. Wu W. Dentchev T. Zeng Y. Wang J. Tsui I.. (2004). Light damage induced changes in mouse retinal gene expression. Exp. Eye Res. 79, 239–247. 10.1016/j.exer.2004.05.002 15325571

[B17] Chi S. W. Zang J. B. Mele A. Darnell R. B. (2009). Argonaute HITS-CLIP decodes microRNA-mRNA interaction maps. Nature 460, 479–486. 10.1038/nature08170 19536157 PMC2733940

[B18] Chu-Tan J. A. Feng Z. Wooff Y. Cioanca A. V. Schuhmann U. Aggio-Bruce R. (2020). Functional microRNA targetome undergoes degeneration-induced shift in the retina. bioRxiv [preprint]. 10.1101/2020.05.27.118307 PMC8406976 34465369

[B19] Chu-Tan J. A. Rutar M. Saxena K. Aggio-Bruce R. Essex R. W. Valter K.. (2018). MicroRNA-124 dysregulation is associated with retinal inflammation and photoreceptor death in the degenerating retina. Invest. Ophthalmol. Vis. Sci. 59, 4094–4105. 10.1167/iovs.18-24623 30098196 PMC11647551

[B20] Danciger M. Matthes M. T. Yasamura D. Akhmedov N. B. Rickabaugh T. Gentleman S.. (2000). A QTL on distal chromosome 3 that influences the severity of light-induced damage to mouse photoreceptors. Mamm. Genome 11, 422–427. 10.1007/s003350010081 10818205

[B21] de Raad S. Szczesny P. J. Munz K. Reme C. E. (1996). Light damage in the rat retina: glial fibrillary acidic protein accumulates in Muller cells in correlation with photoreceptor damage. Ophthalmic Res. 28, 99–107. 10.1159/000267881 8792360

[B22] Deliyanti D. Lee J. Y. Petratos S. Meyer C. J. Ward K. W. Wilkinson-Berka J. L.. (2016). A potent Nrf2 activator, dh404, bolsters antioxidant capacity in glial cells and attenuates ischaemic retinopathy. Clin. Sci. 130, 1375–1387. 10.1042/CS20160068 27005782

[B23] Diaz Quiroz J. F. Tsai E. Coyle M. Sehm T. Echeverri K. (2014). Precise control of miR-125b levels is required to create a regeneration-permissive environment after spinal cord injury: a cross-species comparison between salamander and rat. Dis. Model. Mech. 7, 601–611. 10.1242/dmm.014837 24719025 PMC4036468

[B24] Elsaeidi F. Macpherson P. Mills E. A. Jui J. Flannery J. G. Goldman D. (2018). Notch suppression collaborates with Ascl1 and Lin28 to unleash a regenerative response in fish retina, but not in mice. J. Neurosci. 38, 2246–2261. 10.1523/JNEUROSCI.2126-17.2018 29378863 PMC5830513

[B25] Fausett B. V. Gumerson J. D. Goldman D. (2008). The proneural basic helix-loop-helix gene ascl1a is required for retina regeneration. J. Neurosci. 28, 1109–1117. 10.1523/JNEUROSCI.4853-07.2008 18234889 PMC2800945

[B26] Fischer A. J. Bongini R. (2010). Turning Muller glia into neural progenitors in the retina. Mol. Neurobiol. 42, 199–209. 10.1007/s12035-010-8152-2 21088932

[B27] Geiss G. K. Bumgarner R. E. Birditt B. Dahl T. Dowidar N. Dunaway D. L.. (2008). Direct multiplexed measurement of gene expression with color-coded probe pairs. Nat. Biotechnol. 26, 317–325. 10.1038/nbt1385 18278033

[B28] Goldman D. (2014). Müller glial cell reprogramming and retina regeneration. Nat Rev. Neurosci. 15, 431–442. 10.1038/nrn3723 24894585 PMC4249724

[B29] Gosbell A. D. Stefanovic N. Scurr L. L. Pete J. Kola I. Favilla I.. (2006). Retinal light damage: structural and functional effects of the antioxidant glutathione peroxidase-1. Invest. Ophthalmol. Vis. Sci. 47, 2613–2622. 10.1167/iovs.05-0962 16723478

[B30] Grimm C. Reme C. E. (2013). Light damage as a model of retinal degeneration. Methods Mol. Biol. 935, 87–97. 10.1007/978-1-62703-080-9_6 23150362

[B31] Grosche A. Hauser A. Lepper M. F. Mayo R. von Toerne C. Merl-Pham J.. (2016). The proteome of native adult muller glial cells from murine retina. Mol. Cell Proteomics 15, 462–480. 10.1074/mcp.M115.052183 26324419 PMC4739667

[B32] Grosche J. Hartig W. Reichenbach A. (1995). Expression of glial fibrillary acidic protein (GFAP), glutamine synthetase (GS), and Bcl-2 protooncogene protein by Muller (glial) cells in retinal light damage of rats. Neurosci. Lett. 185, 119–122. 10.1016/0304-3940(94)11239-F 7746501

[B33] Guillonneau X. Regnier-Ricard F. Laplace O. Jonet L. Bryckaert M. Courtois Y.. (1998). Fibroblast growth factor (FGF) soluble receptor 1 acts as a natural inhibitor of FGF2 neurotrophic activity during retinal degeneration. Mol. Biol. Cell 9, 2785–2802. 10.1091/mbc.9.10.2785 9763444 PMC25554

[B34] Gunzburg M. J. Sivakumaran A. Pendini N. R. Yoon J. H. Gorospe M. Wilce M. C.. (2015). Cooperative interplay of let-7 mimic and HuR with MYC RNA. Cell Cycle 14, 2729–2733. 10.1080/15384101.2015.1069930 26177105 PMC4612438

[B35] Gurtan A. M. Sharp P. A. (2013). The role of miRNAs in regulating gene expression networks. J. Mol. Biol. 425, 3582–3600. 10.1016/j.jmb.2013.03.007 23500488 PMC3757117

[B36] Hackler L. Jr. Wan J. Swaroop A. Qian J. Zack D. J. (2010). MicroRNA profile of the developing mouse retina. Invest. Ophthalmol. Vis. Sci. 51, 1823–1831. 10.1167/iovs.09-4657 19933188 PMC2868396

[B37] Hai T. Jalgaonkar S. Wolford C. C. Yin X. (2011). Immunohistochemical detection of activating transcription factor 3, a hub of the cellular adaptive–response network. Meth. Enzymol. 490, 175–194. 10.1016/B978-0-12-385114-7.00011-8 21266251 PMC3675787

[B38] Hara E. S. Ono M. Eguchi T. Kubota S. Pham H. T. Sonoyama W.. (2013). miRNA-720 controls stem cell phenotype, proliferation and differentiation of human dental pulp cells. PLoS ONE 8:e83545. 10.1371/journal.pone.0083545 24386225 PMC3875457

[B39] Hartig W. Grosche J. Distler C. Grimm D. el-Hifnawi E. Reichenbach A. (1995). Alterations of Muller (glial) cells in dystrophic retinae of RCS rats. J. Neurocytol. 24, 507–517. 10.1007/BF01179976 7561959

[B40] Hitchcock P. Ochocinska M. Sieh A. Otteson D. (2004). Persistent and injury-induced neurogenesis in the vertebrate retina. Prog. Retin. Eye Res. 23, 183–194. 10.1016/j.preteyeres.2004.01.001 15094130

[B41] Hoang T. Wang J. Boyd P. Wang F. Santiago C. Jiang L.. (2020). Gene regulatory networks controlling vertebrate retinal regeneration. Science 370:eabb8598. 10.1126/science.abb8598 33004674 PMC7899183

[B42] Hong P. Jiang M. Li H. (2014). Functional requirement of dicer1 and miR-17-5p in reactive astrocyte proliferation after spinal cord injury in the mouse. Glia 62, 2044–2060. 10.1002/glia.22725 25043492

[B43] Huyghe A. Van den Ackerveken P. Sacheli R. Prevot P. P. Thelen N. Renauld J.. (2015). MicroRNA-124 regulates cell specification in the cochlea through modulation of Sfrp4/5. Cell Rep. 13, 31–42. 10.1016/j.celrep.2015.08.054 26387953

[B44] Illumina_Inc. (2011). RNA-Seq Data Comparison with Gene Expression Microarrays, a Cross-Platform Comparison of Differential Gene Expression Analysis. San Diego, CA: Illumina White Paper.

[B45] Inoue Y. Shimazawa M. Noda Y. Nagano R. Otsuka T. Kuse Y.. (2017). RS9, a novel Nrf2 activator, attenuates light-induced death of cells of photoreceptor cells and Muller glia cells. J. Neurochem. 141, 750–765. 10.1111/jnc.14029 28345128

[B46] Jeon C. J. Strettoi E. Masland R. H. (1998). The major cell populations of the mouse retina. J. Neurosci. 18, 8936–8946. 10.1523/JNEUROSCI.18-21-08936.1998 9786999 PMC6793518

[B47] Jones B. W. Marc R. E. (2005). Retinal remodeling during retinal degeneration. Exp. Eye Res. 81, 123–137. 10.1016/j.exer.2005.03.006 15916760

[B48] Jones B. W. Watt C. B. Frederick J. M. Baehr W. Chen C. K. Levine E. M.. (2003). Retinal remodeling triggered by photoreceptor degenerations. J. Comp. Neurol. 464, 1–16. 10.1002/cne.10703 12866125

[B49] Jorstad N. L. Wilken M. S. Todd L. Finkbeiner C. Nakamura P. Radulovich N.. (2020). STAT signaling modifies Ascl1 chromatin binding and limits neural regeneration from muller glia in adult mouse retina. Cell Rep. 30, 2195–2208 e2195. 10.1016/j.celrep.2020.01.075 32075759 PMC7148114

[B50] Karagkouni D. Paraskevopoulou M. D. Chatzopoulos S. Vlachos I. S. Tastsoglou S. Kanellos I.. (2018). DIANA-TarBase v8: a decade-long collection of experimentally supported miRNA-gene interactions. Nucleic Acids Res. 46, D239–D245. 10.1093/nar/gkx1141 29156006 PMC5753203

[B51] Karali M. Manfredi A. Puppo A. Marrocco E. Gargiulo A. Allocca M.. (2011). MicroRNA-restricted transgene expression in the retina. PLoS ONE 6:e22166. 10.1371/journal.pone.0022166 21818300 PMC3144214

[B52] Karali M. Peluso I. Marigo V. Banfi S. (2007). Identification and characterization of microRNAs expressed in the mouse eye. Invest. Ophthalmol. Vis. Sci. 48, 509–515. 10.1167/iovs.06-0866 17251443

[B53] Karl M. O. Reh T. A. (2010). Regenerative medicine for retinal diseases: activating endogenous repair mechanisms. Trends Mol. Med. 16, 193–202. 10.1016/j.molmed.2010.02.003 20303826 PMC2854262

[B54] Kassen S. C. Ramanan V. Montgomery J. E. C T.B. Liu C. G. Vihtelic T. S.. (2007). Time course analysis of gene expression during light-induced photoreceptor cell death and regeneration in albino zebrafish. Dev. Neurobiol. 67, 1009–1031. 10.1002/dneu.20362 17565703

[B55] Kim H. H. Kuwano Y. Srikantan S. Lee E. K. Martindale J. L. Gorospe M. (2009). HuR recruits let-7/RISC to repress c-Myc expression. Genes Dev. 23, 1743–1748. 10.1101/gad.1812509 19574298 PMC2720259

[B56] Kim K. H. Jeong J. Y. Surh Y. J. Kim K. W. (2010). Expression of stress-response ATF3 is mediated by Nrf2 in astrocytes. Nucleic Acids Res. 38, 48–59. 10.1093/nar/gkp865 19864258 PMC2800224

[B57] Kole C. Brommer B. Nakaya N. Sengupta M. Bonet-Ponce L. Zhao T.. (2020). Activating transcription factor 3 (ATF3) protects retinal ganglion cells and promotes functional preservation after optic nerve crush. Invest. Ophthalmol. Vis. Sci. 61:31. 10.1167/iovs.61.2.31 32084268 PMC7326601

[B58] Ku H. C. Cheng C. F. (2020). Master regulator activating transcription factor 3 (ATF3) in metabolic homeostasis and cancer. Front. Endocrinol. (Lausanne) 11:556. 10.3389/fendo.2020.00556 32922364 PMC7457002

[B59] Kuhrt H. Wurm A. Karl A. Iandiev I. Wiedemann P. Reichenbach A.. (2008). Muller cell gliosis in retinal organ culture mimics gliotic alterations after ischemia in vivo. Int. J. Dev. Neurosci 26, 745–751. 10.1016/j.ijdevneu.2008.07.003 18672046

[B60] La Torre A. Georgi S. Reh T. A. (2013). Conserved microRNA pathway regulates developmental timing of retinal neurogenesis. Proc. Natl. Acad. Sci. U.S.A. 110, E2362–2370. 10.1073/pnas.1301837110 23754433 PMC3696811

[B61] Lanz T. A. Reinhart V. Sheehan M. J. Rizzo S. J. S. Bove S. E. James L. C. (2019). Postmortem transcriptional profiling reveals widespread increase in inflammation in schizophrenia: a comparison of prefrontal cortex, striatum, and hippocampus among matched tetrads of controls with subjects diagnosed with schizophrenia, bipolar or major depressive disorder. Transl. Psychiatry 9:151 10.1038/s41398-019-0492-8 31123247 PMC6533277

[B62] Lee H. Han S. Kwon C. S. Lee D. (2016). Biogenesis and regulation of the let-7 miRNAs and their functional implications. Protein Cell 7, 100–113. 10.1007/s13238-015-0212-y 26399619 PMC4742387

[B63] Lee S. T. Chu K. Oh H. J. Im W. S. Lim J. Y. Kim S. K.. (2011). Let-7 microRNA inhibits the proliferation of human glioblastoma cells. J. Neurooncol. 102, 19–24. 10.1007/s11060-010-0286-6 20607356

[B64] Lee Y. S. Dutta A. (2007). The tumor suppressor microRNA let-7 represses the HMGA2 oncogene. Genes Dev. 21, 1025–1030. 10.1101/gad.1540407 17437991 PMC1855228

[B65] Lenkowski J. R. Raymond P. A. (2014). Müller glia: stem cells for generation and regeneration of retinal neurons in teleost fish. Prog. Retin. Eye Res. 40, 94–123. 10.1016/j.preteyeres.2013.12.007 24412518 PMC3999222

[B66] Leppert U. Henke W. Huang X. Muller J. M. Dubiel W. (2011). Post-transcriptional fine-tuning of COP9 signalosome subunit biosynthesis is regulated by the c-Myc/Lin28B/let-7 pathway. J. Mol. Biol. 409, 710–721. 10.1016/j.jmb.2011.04.041 21530537

[B67] Leung A. K. L. (2015). The Whereabouts of microRNA Actions: cytoplasm and Beyond. Trends Cell Biol. 25, 601–610. 10.1016/j.tcb.2015.07.005 26410406 PMC4610250

[B68] Li L. Z. Zhang C. Z. Liu L. L. Yi C. Lu S. X. Zhou X.. (2014). miR-720 inhibits tumor invasion and migration in breast cancer by targeting TWIST1. Carcinogenesis 35, 469–478. 10.1093/carcin/bgt330 24085799

[B69] Liebermann D. A. Hoffman B. (2007). Gadd45 in the response of hematopoietic cells to genotoxic stress. Blood Cells Mol. Dis. 39, 329–335. 10.1016/j.bcmd.2007.06.006 17659913 PMC3268059

[B70] Lukiw W. J. (2007). Micro-RNA speciation in fetal, adult and Alzheimer's disease hippocampus. Neuroreport 18, 297–300. 10.1097/WNR.0b013e3280148e8b 17314675

[B71] Luu J. Kallestad L. Hoang T. Lewandowski D. Dong Z. Blackshaw S.. (2020). Epigenetic hallmarks of age-related macular degeneration are recapitulated in a photosensitive mouse model. Hum. Mol. Genet. 29, 2611–2624. 10.1093/hmg/ddaa158 32691052 PMC7471509

[B72] Ma D. K. Jang M. H. Guo J. U. Kitabatake Y. Chang M. L. Pow-Anpongkul N.. (2009). Neuronal activity-induced Gadd45b promotes epigenetic DNA demethylation and adult neurogenesis. Science 323, 1074–1077. 10.1126/science.1166859 19119186 PMC2726986

[B73] Maldotti M. Incarnato D. Neri F. Krepelova A. Rapelli S. Anselmi F.. (2016). The long intergenic non-coding RNA CCR492 functions as a let-7 competitive endogenous RNA to regulate c-Myc expression. Biochim. Biophys. Acta 1859, 1322–1332. 10.1016/j.bbagrm.2016.06.010 27344374

[B74] Mansouri V. Razzaghi M. Rostami-Nejad M. Rezaei-Tavirani M. Heidari M. H. Safari S.. (2020). Neuroprotective properties of photobiomodulation in retinal regeneration in rats: perspectives from interaction levels. J. Lasers Med. Sci. 11, 280–286. 10.34172/jlms.2020.47 32802288 PMC7369552

[B75] Marc R. E. Jones B. W. (2003). Retinal remodeling in inherited photoreceptor degenerations. Mol. Neurobiol 28, 139–147. 10.1385/MN:28:2:139 14576452

[B76] Marc R. E. Jones B. W. Anderson J. R. Kinard K. Marshak D. W. Wilson J. H.. (2007). Neural reprogramming in retinal degeneration. Invest. Ophthalmol. Vis. Sci. 48, 3364–3371. 10.1167/iovs.07-0032 17591910 PMC2408857

[B77] Men Y. Yelick J. Jin S. Tian Y. Chiang M. S. R. Higashimori H.. (2019). Exosome reporter mice reveal the involvement of exosomes in mediating neuron to astroglia communication in the CNS. Nat. Commun. 10:4136. 10.1038/s41467-019-11534-w 31515491 PMC6742670

[B78] Morel L. Regan M. Higashimori H. Ng S. K. Esau C. Vidensky S.. (2013). Neuronal exosomal miRNA-dependent translational regulation of astroglial glutamate transporter GLT1. J. Biol. Chem. 288, 7105–7116. 10.1074/jbc.M112.410944 23364798 PMC3591620

[B79] Naskar R. Thanos S. (2006). Retinal gene profiling in a hereditary rodent model of elevated intraocular pressure. Mol. Vis. 12, 1199–1210. Available online at: http://www.molvis.org/molvis/v12/a13, 17102796

[B80] Natoli R. Zhu Y. Valter K. Bisti S. Eells J. Stone J. (2010). Gene and noncoding RNA regulation underlying photoreceptor protection: microarray study of dietary antioxidant saffron and photobiomodulation in rat retina. Mol. Vis 16, 1801–1822. 20844572 PMC2932490

[B81] Nelson B. R. Ueki Y. Reardon S. Karl M. O. Georgi S. Hartman B. H.. (2011). Genome-wide analysis of Muller glial differentiation reveals a requirement for Notch signaling in postmitotic cells to maintain the glial fate. PLoS ONE 6:e22817. 10.1371/journal.pone.0022817 21829655 PMC3149061

[B82] Newman E. Reichenbach A. (1996). The Muller cell: a functional element of the retina. Trends Neurosci. 19, 307–312. 10.1016/0166-2236(96)10040-0 8843598

[B83] Otteson D. C. (2017). Talkin' about my (re)generation: The who of intrinsic retinal stem cells. Neuroscience 346, 447–449. 10.1016/j.neuroscience.2017.01.022 28131621

[B84] Pogue A. I. Cui J. G. Li Y. Y. Zhao Y. Culicchia F. Lukiw W. J. (2010). Micro RNA-125b (miRNA-125b) function in astrogliosis and glial cell proliferation. Neurosci. Lett. 476, 18–22. 10.1016/j.neulet.2010.03.054 20347935

[B85] Ramachandran R. Fausett B. V. Goldman D. (2010). Ascl1a regulates Müller glia dedifferentiation and retinal regeneration through a Lin-28-dependent, let-7 microRNA signalling pathway. Nat. Cell Biol. 12, 1101–1107. 10.1038/ncb2115 20935637 PMC2972404

[B86] Rao M. S. Van Vleet T. R. Ciurlionis R. Buck W. R. Mittelstadt S. W. Blomme E. A. G.. (2018). Comparison of RNA-Seq and microarray gene expression platforms for the toxicogenomic evaluation of liver from short-term rat toxicity studies. Front. Genet. 9:636. 10.3389/fgene.2018.00636 30723492 PMC6349826

[B87] Rao V. T. Ludwin S. K. Fuh S. C. Sawaya R. Moore C. S. Ho M. K.. (2016). MicroRNA expression patterns in human astrocytes in relation to anatomical location and age. J. Neuropathol. Exp. Neurol. 75, 156–166. 10.1093/jnen/nlv016 26802178

[B88] Rattner A. Nathans J. (2005). The genomic response to retinal disease and injury: evidence for endothelin signaling from photoreceptors to glia. J. Neurosci. 25, 4540–4549. 10.1523/JNEUROSCI.0492-05.2005 15872101 PMC6725023

[B89] Reichenbach A. Bringmann A. (2013). New functions of Muller cells. Glia 61, 651–678. 10.1002/glia.22477 23440929

[B90] Reichenbach A. Stolzenburg J. U. Eberhardt W. Chao T. I. Dettmer D. Hertz L. (1993). What do retinal muller (glial) cells do for their neuronal “small siblings”? J. Chem. Neuroanat. 6, 201–213. 10.1016/0891-0618(93)90042-3 8104418

[B91] Reik W. (2007). Stability and flexibility of epigenetic gene regulation in mammalian development. Nature 447, 425–432. 10.1038/nature05918 17522676

[B92] Rennie W. Kanoria S. Liu C. Mallick B. Long D. Wolenc A.. (2016). STarMirDB: A database of microRNA binding sites. RNA Biol. 13, 554–560. 10.1080/15476286.2016.1182279 27144897 PMC4962797

[B93] Roberts T. C. (2015). The microRNA Machinery. Adv. Exp. Med. Biol. 887, 15–30. 10.1007/978-3-319-22380-3_2 26662984

[B94] Rutar M. Natoli R. Chia R. X. Valter K. Provis J. M. (2015). Chemokine-mediated inflammation in the degenerating retina is coordinated by Muller cells, activated microglia, and retinal pigment epithelium. J. Neuroinflammation 12:8. 10.1186/s12974-014-0224-1 25595590 PMC4308937

[B95] Samardzija M. Wenzel A. Naash M. Reme C. E. Grimm C. (2006). Rpe65 as a modifier gene for inherited retinal degeneration. Eur. J. Neurosci. 23, 1028–1034. 10.1111/j.1460-9568.2006.04639.x 16519667 PMC2823586

[B96] Saul K. E. Koke J. R. Garcia D. M. (2010). Activating transcription factor 3 (ATF3) expression in the neural retina and optic nerve of zebrafish during optic nerve regeneration. Comp. Biochem. Physiol. Part A Mol. Integr. Physiol. 155, 172–182. 10.1016/j.cbpa.2009.10.042 19896551

[B97] Schopman N. C. Heynen S. Haasnoot J. Berkhout B. (2010). A miRNA-tRNA mix-up: tRNA origin of proposed miRNA. RNA Biol. 7, 573–576. 10.4161/rna.7.5.13141 20818168

[B98] Shenoy A. Danial M. Blelloch R. H. (2015). Let-7 and miR-125 cooperate to prime progenitors for astrogliogenesis. EMBO J. 34, 1180–1194. 10.15252/embj.201489504 25715649 PMC4426479

[B99] Sundermeier T. R. Palczewski K. (2012). The physiological impact of microRNA gene regulation in the retina. Cell. Mol. Life Sci. 69, 2739–2750. 10.1007/s00018-012-0976-7 22460583 PMC3443797

[B100] Tan S. M. Deliyanti D. Figgett W. A. Talia D. M. de Haan J. B. Wilkinson-Berka J. L. (2015). Ebselen by modulating oxidative stress improves hypoxia-induced macroglial Muller cell and vascular injury in the retina. Exp. Eye Res. 136, 1–8. 10.1016/j.exer.2015.04.015 25912997

[B101] Tang Y. Lin Y. Li C. Hu X. Liu Y. He M.. (2015). MicroRNA-720 promotes in vitro cell migration by targeting Rab35 expression in cervical cancer cells. Cell Biosci. 5:56. 10.1186/s13578-015-0047-5 26413265 PMC4583841

[B102] Tao Z. Zhao C. Jian Q. Gillies M. Xu H. Yin Z. Q. (2016). Lin28B promotes Muller glial cell de-differentiation and proliferation in the regenerative rat retinas. Oncotarget 7, 49368–49383. 10.18632/oncotarget.10343 27384999 PMC5226514

[B103] Thummel R. Kassen S. C. Montgomery J. E. Enright J. M. Hyde D. R. (2008). Inhibition of Muller glial cell division blocks regeneration of the light-damaged zebrafish retina. Dev. Neurobiol. 68, 392–408. 10.1002/dneu.20596 18161852 PMC3711086

[B104] Tokumaru S. Suzuki M. Yamada H. Nagino M. Takahashi T. (2008). let-7 regulates Dicer expression and constitutes a negative feedback loop. Carcinogenesis 29, 2073–2077. 10.1093/carcin/bgn187 18700235

[B105] Torres-Martin M. Lassaletta L. de Campos J. M. Isla A. Gavilan J. Pinto G. R.. (2013). Global profiling in vestibular schwannomas shows critical deregulation of microRNAs and upregulation in those included in chromosomal region 14q32. PLoS ONE 8:e65868. 10.1371/journal.pone.0065868 23776562 PMC3679163

[B106] Ueki Y. Karl M. O. Sudar S. Pollak J. Taylor R. J. Loeffler K.. (2012). P53 is required for the developmental restriction in Müller glial proliferation in mouse retina. Glia 60, 1579–1589. 10.1002/glia.22377 22777914 PMC3422417

[B107] Ueki Y. Reh T. A. (2012). Activation of BMP-Smad1/5/8 signaling promotes survival of retinal ganglion cells after damage in vivo. PLoS ONE 7:e38690. 10.1371/journal.pone.0038690 22701694 PMC3368846

[B108] Ueki Y. Wang J. Chollangi S. Ash J. D. (2008). STAT3 activation in photoreceptors by leukemia inhibitory factor is associated with protection from light damage. J. Neurochem. 105, 784–796. 10.1111/j.1471-4159.2007.05180.x 18088375

[B109] Vihtelic T. S. Soverly J. E. Kassen S. C. Hyde D. R. (2006). Retinal regional differences in photoreceptor cell death and regeneration in light-lesioned albino zebrafish. Exp. Eye Res. 82, 558–575. 10.1016/j.exer.2005.08.015 16199033

[B110] Vlachos I. S. Zagganas K. Paraskevopoulou M. D. Georgakilas G. Karagkouni D. Vergoulis T.. (2015). DIANA-miRPath v3.0: deciphering microRNA function with experimental support. Nucleic Acids Res. 43, W460–466. 10.1093/nar/gkv403 25977294 PMC4489228

[B111] Wan J. Goldman D. (2016). Retina regeneration in zebrafish. Curr. Opin. Genet. Dev. 40, 41–47. 10.1016/j.gde.2016.05.009 27281280 PMC5135611

[B112] Wang Q. Li W. X. Dai S. X. Guo Y. C. Han F. F. Zheng J. J.. (2017). Meta-Analysis of Parkinson's Disease and Alzheimer's disease revealed commonly impaired pathways and dysregulation of NRF2-dependent genes. J. Alzheimers. Dis. 56, 1525–1539. 10.3233/JAD-161032 28222515

[B113] Wang X. Fan J. Zhang M. Sun Z. Xu G. (2013). Gene expression changes under cyclic mechanical stretching in rat retinal glial (Muller) cells. PLoS ONE 8:e63467. 10.1371/journal.pone.0063467 23723984 PMC3664568

[B114] Wenzel A. Grimm C. Samardzija M. Reme C. E. (2005). Molecular mechanisms of light-induced photoreceptor apoptosis and neuroprotection for retinal degeneration. Prog. Retin. Eye Res. 24, 275–306. 10.1016/j.preteyeres.2004.08.002 15610977

[B115] Wenzel A. Reme C. E. Williams T. P. Hafezi F. Grimm C. (2001). The Rpe65 Leu450Met variation increases retinal resistance against light-induced degeneration by slowing rhodopsin regeneration. J. Neurosci. 21, 53–58. 10.1523/JNEUROSCI.21-01-00053.2001 11150319 PMC6762429

[B116] Winkler B. S. Boulton M. E. Gottsch J. D. Sternberg P. (1999). Oxidative damage and age-related macular degeneration. Mol. Vis. 5:32. 10562656 PMC1773059

[B117] Wohl S. G. Jorstad N. L. Levine E. M. Reh T. A. (2017). Muller glial microRNAs are required for the maintenance of glial homeostasis and retinal architecture. Nat. Commun. 8:1603. 10.1038/s41467-017-01624-y 29150673 PMC5693933

[B118] Wohl S. G. Reh T. A. (2016). The microRNA expression profile of mouse Muller glia in vivo and in vitro. Sci. Rep. 6:35423. 10.1038/srep35423 27739496 PMC5064377

[B119] Wong T. S. Man O. Y. Tsang C. M. Tsao S. W. Tsang R. K. Chan J. Y.. (2011). MicroRNA let-7 suppresses nasopharyngeal carcinoma cells proliferation through downregulating c-Myc expression. J. Cancer Res. Clin. Oncol 137, 415–422. 10.1007/s00432-010-0898-4 20440510 PMC3036828

[B120] Wooff Y. Cioanca A. V. Chu-Tan J. A. Aggio-Bruce R. Schumann U. Natoli R. (2020). Small-medium extracellular vesicles and their miRNA cargo in retinal health and degeneration: mediators of homeostasis, and vehicles for targeted gene therapy. Front. Cell. Neurosci 14:160. 10.3389/fncel.2020.00160 32670023 PMC7330137

[B121] Xue W. Cojocaru R. I. Dudley V. J. Brooks M. Swaroop A. Sarthy V. P. (2011). Ciliary neurotrophic factor induces genes associated with inflammation and gliosis in the retina: a gene profiling study of flow-sorted, Muller cells. PLoS ONE 6:e20326. 10.1371/journal.pone.0020326 21637858 PMC3102695

[B122] Yang P. Agapova O. Parker A. Shannon W. Pecen P. Duncan J.. (2004). DNA microarray analysis of gene expression in human optic nerve head astrocytes in response to hydrostatic pressure. Physiol. Genomics 17, 157–169. 10.1152/physiolgenomics.00182.2003 14747662

[B123] Yurco P. Cameron D. A. (2005). Responses of Muller glia to retinal injury in adult zebrafish. Vision Res. 45, 991–1002. 10.1016/j.visres.2004.10.022 15695184

[B124] Zamanian J. L. Xu L. Foo L. C. Nouri N. Zhou L. Giffard R. G.. (2012). Genomic analysis of reactive astrogliosis. J. Neurosci. 32, 6391–6410. 10.1523/JNEUROSCI.6221-11.2012 22553043 PMC3480225

[B125] Zuzic M. Rojo Arias J. E. Wohl S. G. Busskamp V. (2019). Retinal miRNA Functions in Health and Disease. Genes (Basel) 10:377. 10.3390/genes10050377 31108959 PMC6562649

