# Supplementary material for: A Comparative Analysis of Reactive Müller Glia Gene Expression After Light Damage and microRNA-Depleted Müller Glia—Focus on microRNAs
Source: Front Cell Dev Biol. 2021 Jan 26;8:620459. doi: 10.3389/fcell.2020.620459 (PMC7891663; doi:10.3389/fcell.2020.620459)
Supplement: Supplementary file 1 [file Data_Sheet_1.pdf]

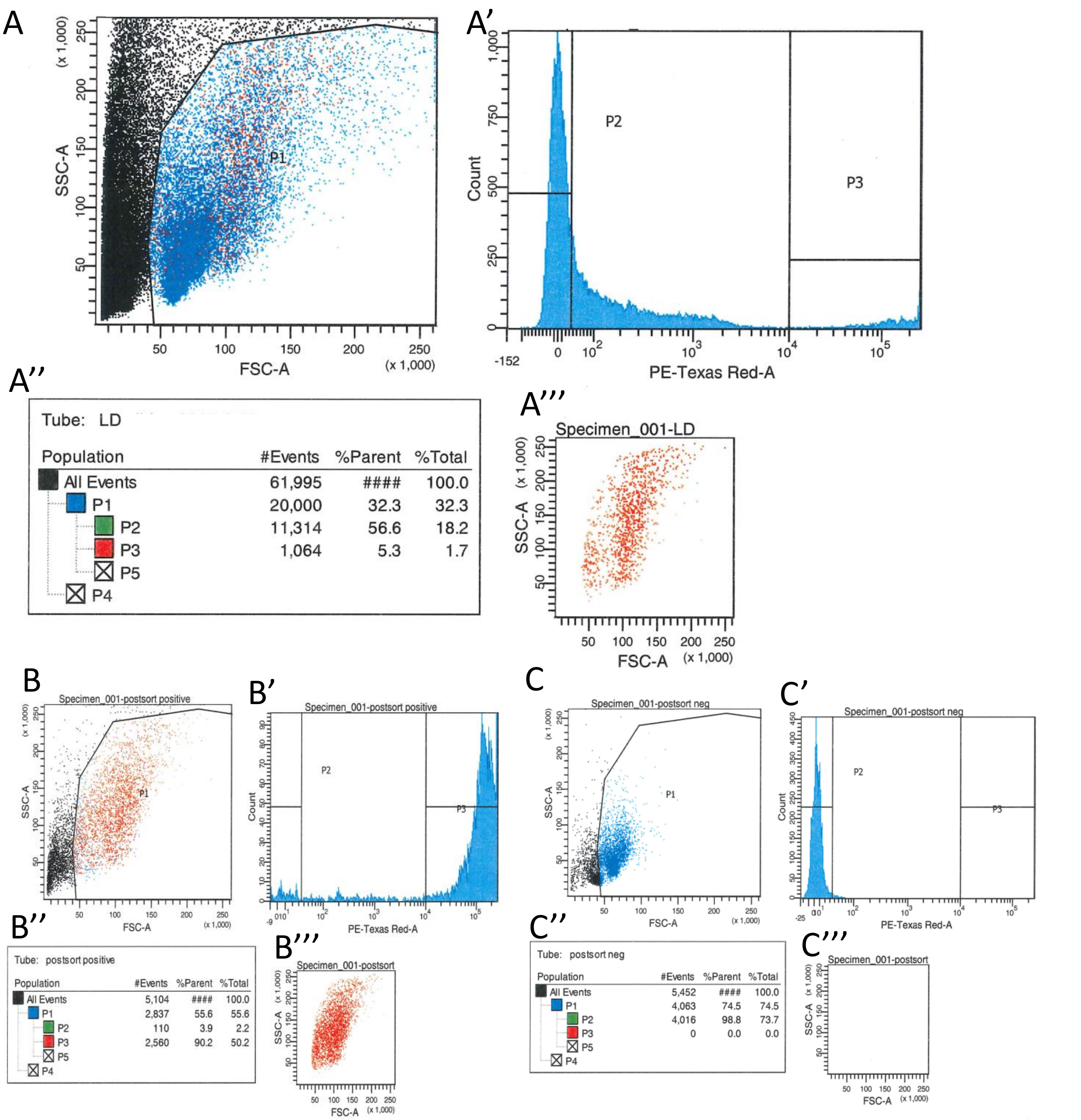

Supplementary Figure 1: Pattern and distribution of light damaged retinas during Fluorescence-activated cell sorting (FACS) to isolate Müller glia (MG). A-A''': All cells in gate P1 (blue dots) have been sorted based on their size (forward scattered, FSC) and fluorescence/ granularity (side scattered, SSC). Very small cells and debris (black dots) have been excluded. tdTomato^{+} cells were found in gate P3 (brightest fluorescence), while the vast majority of tdTomato^{-} cells were found in gate P2 (no fluorescence, A'). A'': Percentage of the P2 and P3. A''': Fraction of sorted tdTomato^{+} cells in the SSC and FSC-A scheme. **B-B'''**: Post-sort of MG fraction (P3 from A') to confirm purity. All cells in gate P1 were post-sorted (B). B': Vast majority of cells are in Gate P3 with high fluorescence. B'': Fraction of cells from MG post-sort. B''': Distribution patterns from MG post-sort in gate 3 (MG gate). **C-C'''**: Post-sort of tdTomato negative fraction (P2 from A') to confirm purity. All cells in gate P1 were post-sorted (C). C': Vast majority of cells are in gate P2 with no fluorescence. C'': Fraction of cells from tdTomato negative post-sort. C''': Distribution patterns from tdTomato negative post-sort in gate 3 (MG gate).

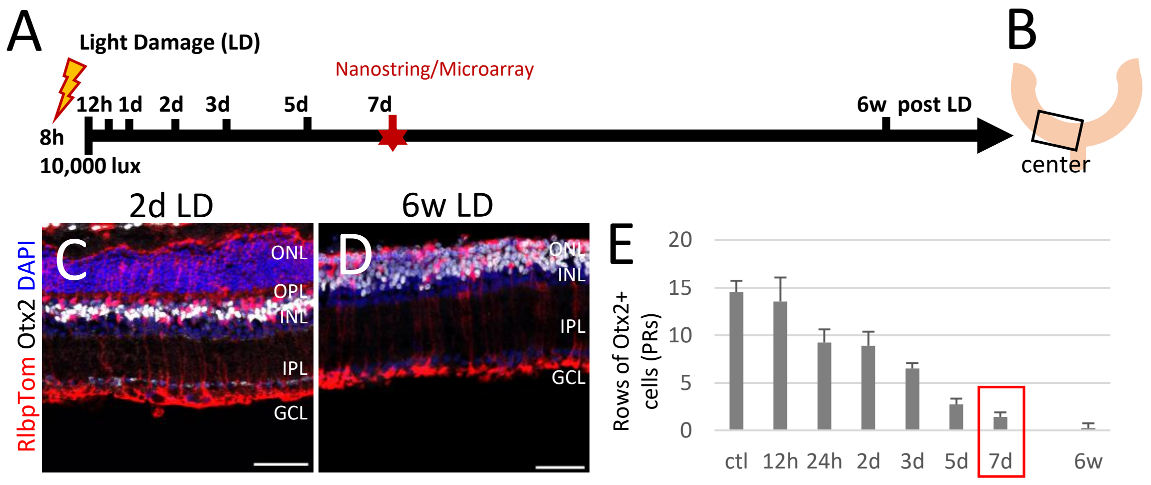

Supplementary Figure 2: Time course of photoreceptor loss in the central retina after light damage (LD). A: Experimental design. **B**: Regions of analysis. **C-D:** Immunofluorescence labeling for tdTomato (MG), Otx2, and DAPI nuclear staining of the center retina 2 days and 6 weeks after light damage (LD). **E:** Number of Otx2+ photoreceptor (PR) rows in the ONL at all time points of analysis. Scale bars 50 µm. ONL: outer nuclear layer, OPL: outer plexiform layer, INL: inner nuclear layer, IPL: inner plexiform layer, GCL: ganglion cell layer, LD: light damage.

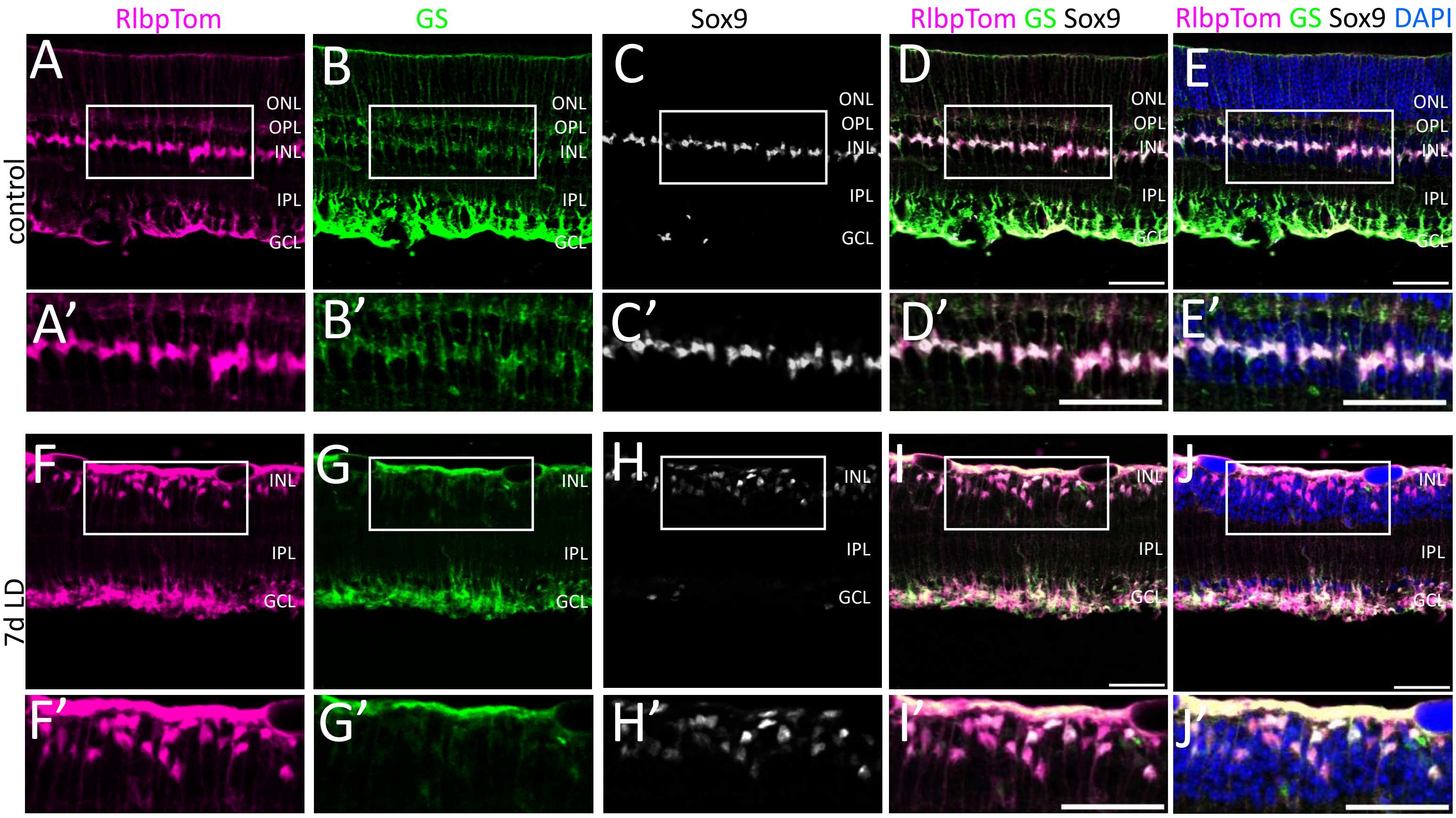

Supplementary Figure 3: GFAP and glutamine synthetase expression. A/A'-J/J': Immunofluorescence labeling for tdTomato (MG), GS (Glutamine synthetase), and Sox9 of the center retina of an undamaged control (A/A'-E/E') and 7 days after light damage (LD, F/F'-J/J'). Scale bars 50 µm. ONL: outer nuclear layer, OPL: outer plexiform layer, INL: inner nuclear layer, IPL: inner plexiform layer, GCL: ganglion cell layer, LD: light damage.

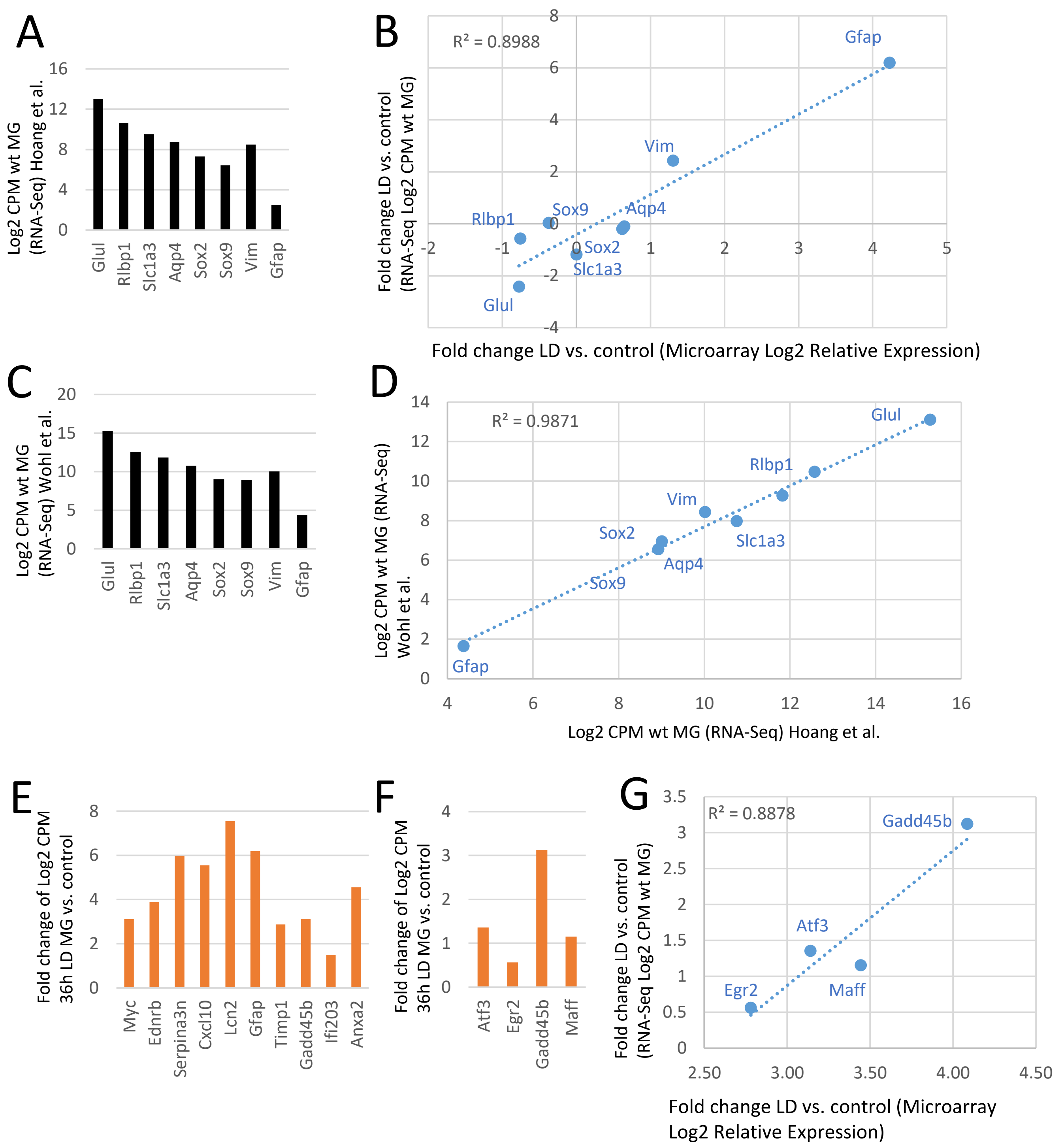

Supplementary Figure 4: Glial gene expression of Müller glia in undamaged retinas and after light damage (LD). A: Log2 of counts per million (CPM, RNA-Seq) of glial genes in normal MG of adult mice (dataset from Hoang et al., 2020, 4 technical replicates, GlastCreERT-GFP mouse). **B:** Correlation of MG gene expression (fold change light damaged versus control) of microarray (7d LD) and RNA-Seq dataset (36h LD). **C:** Log2 of counts per million (CPM, RNA-Seq) of glial genes in normal MG of adult mice (dataset from Wohl et al., 2017, one technical replicate, Rlbp1CreERT-tdTomato mouse). **D:** Correlation of MG gene expression of RNA-Seq datasets from Hoang et al., 2020 and Wohl at al., 2017 (4 and 1 technical replicates respectively). **E:** Expression levels of top 10 highly upregulated genes (from Figure 4C) 36h post LD. **F**: Expression levels of identified stress genes upregulated 7 days after LD and 1 month after Dicer-cKO (Figure 6B,C) 36h post LD. **G:** Correlation gene expression levels (fold change LD versus control) of *Egr2*, *Atf3*, *Maff* and *Gadd45b* 7d after LD (microarray) and 36h after LD (RNA-Seq). The dataset of 36h LD used in C-G is from Hoang et al., 2020.

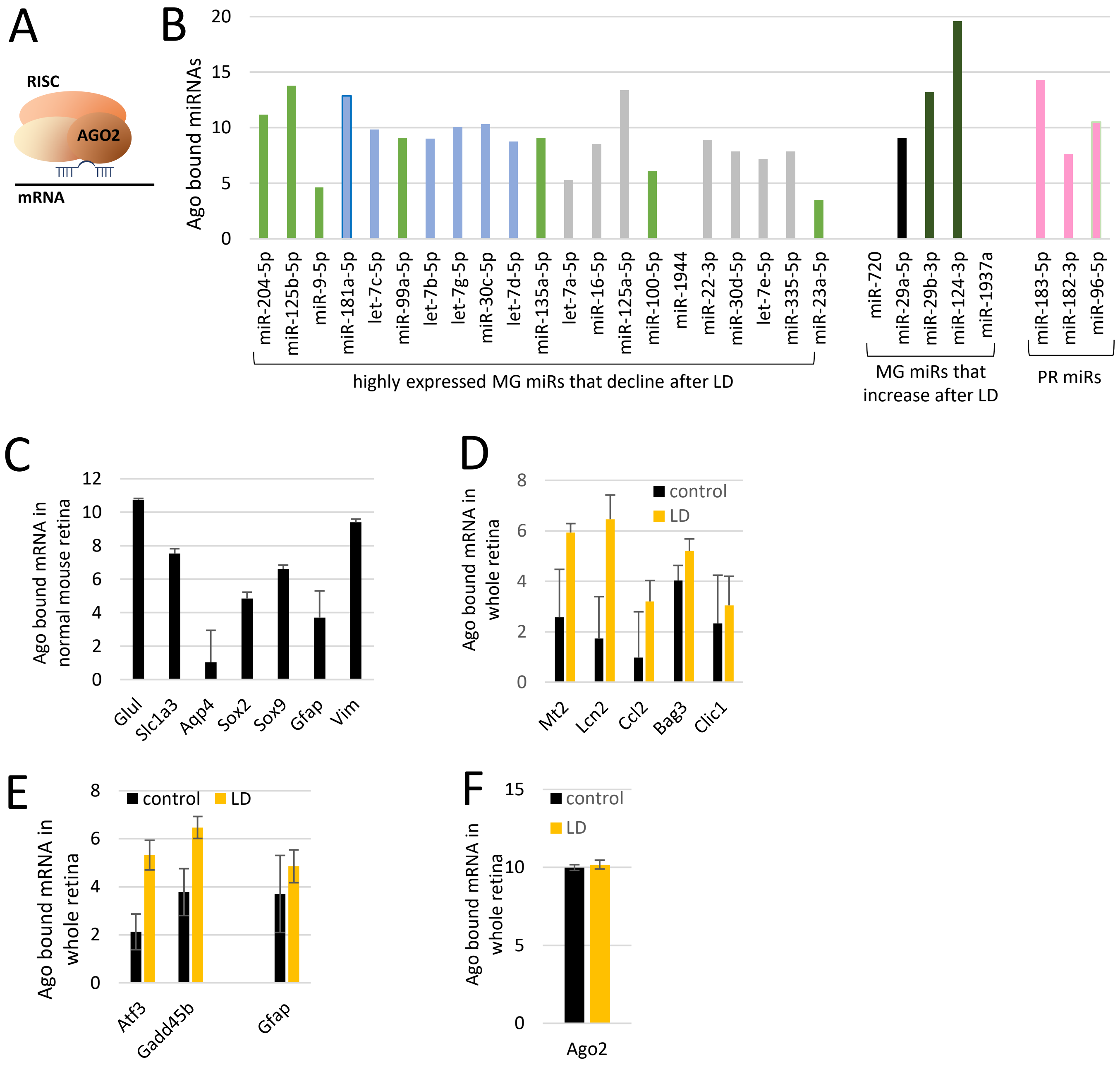

Supplementary Figure 5: Ago-bound miRNAs and mRNAs in whole normal retina and after light damage (LD). A: Schematic of RISC (RNA-induced silencing complex) with Ago2 (Argonaute 2), bound miRNA, and target mRNA. **B:** Ago HITS-CLIP data with Ago2-bound miRNAs found in whole retinas. These include all miRNAs expressed in MG (mGliomiRs, shared miRs and others) as well as the miRNAs that increased after light damage and photoreceptor miRNAs. **C:** Glial gene mRNA found to be Ago-bound in normal retinas. **D:** Genes highly expressed in MG after light damage that are Ago2-bound. **E:** Genes found to be upregulated after both, light damage and Dicer deletion in MG that are Ago2-bound. **F:** Ago2 mRNA is bound to Ago2 with no differences in expression after light damage. Dataset from Chu-Tan et al., 2020, ctl: n=4, light damage: n=4.
