# Supplementary material for: A Comparative Analysis of Reactive Müller Glia Gene Expression After Light Damage and microRNA-Depleted Müller Glia—Focus on microRNAs
Source: Front Cell Dev Biol. 2021 Jan 26;8:620459. doi: 10.3389/fcell.2020.620459 (PMC7891663; doi:10.3389/fcell.2020.620459)
Supplement: Supplementary file 2 [file Data_Sheet_2.pdf]

Supplementary Tables

Supplementary Table 1: Genotyping Primers

**Gene name**	**Forward sequence (5' to 3')**	**Reverse sequence (3' to 5')**
Rlbp1Cre transgene	CAA GTG TGA GAG ACA GCA TTG	TCC TTA GCG CCG TAA ATC AA
tdTomato wildtype	AAG GGA GCT GCA GTG GAG TA	CCG AAA ATC TGT GGG AAG TC
tdTomato mutant	CTG TTC CTG TAC GGC ATG G	GGC ATT AAA GCA GCG TAT CC
RPE65	CAC TGT GGT CTC TGC TAT CTT C	GGT GCA GTT CCA CTT CAG TT

Supplementary Table 2: Primary Antibodies

**Antibody**	**Concentration**	**Company, Catalog #**
rat anti RFP (tdTomato)	1:500	Antibodies online, ABIN334653
mouse anti glutamine synthetase (GS)	1:200	Millipore, MAB302
rabbit anti Sox9	1:1000	Millipore, AB5535
rabbit anti GFAP	1:1000	Dako, Z033401-2
goat anti Otx2	1:250	R&D Systems, AF1979

Supplementary Table 3. Glial gene expression in normal and light damaged Müller glia (Microarray).

Gene Symbol	Undamaged control log2 relative expression	7 days after light damage log2 relative expression	Fold change light damage versus control
Glul	13.84596	13.06892	-0.78
Rlbp1	12.38061	11.62174	-0.76
Aqp4	11.80212	11.42555	-0.38
Vim	11.88869	13.19273	1.30
Slc1a3	12.14886	12.14886	0.00
Sox9	10.11897	10.73825	0.62
Sox2	10.94028	11.58379	0.64
Gfap	6.282722	10.5111	4.23

Supplementary Table 4: Expression levels (decline) of the Müller glia (MG) miRNAs (cutoff 3000 counts) after light damage (LD), in normal adult wild type and Dicer-CKO MG (Nanostring).

Probe Name	normal MG (counts)	MG Light damage (counts)	Dicer-CKO MG (counts)	% decline after LD	%decline after cKO	Accession #
mmu-miR-204	285706	51963	30438	-82	-89	MIMAT0000237
mmu-miR-125b-5p	109596	19063	18202	-83	-83	MIMAT0000136
mmu-miR-9	75416	38870	18100	-48	-76	MIMAT0000142
mmu-miR-181a	48829	19965	8300	-59	-83	MIMAT0000210
mmu-let-7c	26178	9888	9984	-62	-62	MIMAT0000523
mmu-miR-99a	19815	9626	2284	-51	-88	MIMAT0000131
mmu-let-7b	18043	11425	3875	-37	-79	MIMAT0000522
mmu-let-7g	15643	13871	6952	-11	-56	MIMAT0000121
mmu-miR-30c	13955	7419	3296	-47	-76	MIMAT0000514
mmu-let-7d	13294	6634	3557	-50	-73	MIMAT0000383
mmu-miR-135a	12833	11626	4865	-9	-62	MIMAT0000147
mmu-let-7a	8129	3648	2704	-55	-67	MIMAT0000521
mmu-miR-16	7260	2732	3358	-62	-54	MIMAT0000527
mmu-miR-125a-5p	5639	1275	661	-77	-88	MIMAT0000135
mmu-miR-100	5626	1507	524	-73	-91	MIMAT0000655
mmu-miR-1944	5024	2622	3535	-48	-30	MIMAT0009409

mmu-miR-22	4916	4438	1236	-10	-75	MIMAT0000531
mmu-miR-30d	4676	1910	909	-59	-81	MIMAT0000515
mmu-let-7e	3790	1521	984	-60	-74	MIMAT0000524
mmu-miR-335-5p	3329	1702	1311	-49	-61	MIMAT0000766
mmu-miR-23a	3327	1796	1515	-46	-54	MIMAT0000532

Supplementary Table 5: Expression levels (increase) of the Müller glia (MG) miRNAs after light damage (LD, cutoff 5000 counts after LD), in normal adult wild type and Dicer-CKO MG (Nanostring).

Probe Name	normal MG (counts)	MG Light damage (counts)	Dicer-CKO MG (counts)	Fold increase after LD	Fold incerase cKO	Accession #
miR-720**	23904	172827	10983	7.2	0.5	MIMAT0003484
miR-29a	8716	10969	4052	1.3	0.5	MIMAT0000535
miR-29b	1748	7515	2005	4.3	1.1	MIMAT0000127
miR-124	1354	5506	12106	4.1	8.9	MIMAT0000134
miR-1937a	1094	6129	1298	5.6	1.2	MIMAT0009401

** The sequence annotated as miR-720 is likely to be a fragment of a tRNA, and therefore is removed from the miRBase database (Schopman et al., 2010).

Supplementary Table 6: Expression levels of Argonaute2-bound miRNAs in whole mouse retinas (data set from the Natoli lab (Chu-Tan et al., 2020)).

Probe Name	Log2CPM light damage		Probe Name	Log2CPM light damage
miR-204-5p	11.17327		miR-1944	-
miR-125b-5p	13.76099		miR-22-3p	8.905651
miR-9-5p	4.631367		miR-30d-5p	7.862324
miR-181a-5p	12.86331		let-7e-5p	7.14469
let-7c-5p	9.809521		miR-335-5p	7.864721
miR-99a-5p	9.097142		miR-23a-5p	3.497926
let-7b-5p	9.04164		miR-720	-
let-7g-5p	10.04702		miR-29a-5p	9.068966
miR-30c-5p	10.32144		miR-29b-3p	13.17489
let-7d-5p	8.755262		miR-124-3p	19.58201
miR-135a-5p	9.069131		miR-1937a	-
let-7a-5p	5.292606		miR-183-5p	14.30006
miR-16-5p	8.524132		miR-182-3p	7.636908
miR-125a-5p	13.37126		miR-96-5p	10.51531
miR-100-5p	6.124773		miR-22-3p	8.905651

Supplementary Table 7: Top 50 genes upregulated after light damage (Microarray).

**Gene**	**Undamaged control log2 relative expression**	**Light Damage log2 relative expression**	**Fold change light damage versus control**
Myc	5.546965	10.58033	5.03
Ednrb	7.38606	12.32283	4.94
Serpina3n	7.027112	11.8461	4.82
Cxcl10	5.769135	10.57737	4.81
Lcn2	7.960248	12.71421	4.75
Gfap	6.282722	10.5111	4.23
Timp1	6.105936	10.3307	4.22
Gadd45b	7.56354	11.64912	4.09
Ifi203	4.298881	8.316713	4.02
Anxa2	5.3885	9.285599	3.90
Steap4	6.341545	10.20562	3.86
Mt2	8.264072	12.11129	3.85
Klf6	8.065225	11.78304	3.72
Ccl2	6.402283	10.11604	3.71
S100a6	6.641229	10.29619	3.65
Tubb6	6.196977	9.784472	3.59
Gbp2	6.841433	10.37846	3.54

Hmox1	6.483181	10.01682	3.53
Acsl5	5.492249	9.016914	3.52
Emp1	6.499097	10.00783	3.51
Chl1	7.436919	10.9089	3.47
Maff	5.317573	8.760696	3.44
Penk	6.833021	10.2276	3.39
Akap12	7.17374	10.51307	3.34
Rnd3	7.763508	11.06458	3.30
Il1rap	6.830734	10.12385	3.29
Nupr1	7.160269	10.37722	3.22
Bace2	6.047527	9.25269	3.21
Rsad2	6.317559	9.501802	3.18
Ptgs2	5.797745	8.972202	3.17
Aldh1a3	6.87675	10.044	3.17
Atf3	8.045691	11.1855	3.14
Cdh13	7.377244	10.51443	3.14
Lgals1	7.972818	11.10459	3.13
Fosl1	6.344384	9.40595	3.06
Tagln2	5.956065	8.991003	3.03
Phlda1	6.358819	9.392575	3.03
Gap43	8.421201	11.44139	3.02

Ifitm3	8.424528	11.38966	2.97
Lif	6.396519	9.344319	2.95
Slc7a11	7.8706	10.81352	2.94
Osmr	8.618423	11.55899	2.94
Ahnak2	5.90714	8.836987	2.93
Cd44	7.835063	10.75019	2.92
Layn	5.905998	8.788326	2.88
Kitl	5.588583	8.422109	2.83
Nes	6.868566	9.689172	2.82
Trp53	6.800496	9.614729	2.81
Hbegf	7.970807	10.77976	2.81
Gas5	7.477266	10.28387	2.81
Egr2	7.160723	9.940983	2.78

Supplementary Table 8. Gene expression (Microarray) of genes reported to be expressed in light damaged retinas or MG of light damaged retinas

Gene Symbol	Description	Undamaged control log2 relative expression	Light Damage log2 relative expression	Fold change light damage versus control
Bcl2	B cell leukemia/lymphoma 2	6.964325	7.039897	0.1
Ctsd	cathepsin D	11.26174	11.74302	0.5
Kcnj10	potassium inwardly-rectifying channel, subfamily J, member 10	10.01217	10.01217	0.0
Ngfr p75	nerve growth factor receptor (TNFR superfamily, member 16)	9.180977	10.22241	1.0
Fgf2	fibroblast growth factor 2	7.965034	9.540471	1.6
Ntf3	neurotrophin 3	6.209656	6.530434	0.3
Ntrk3	neurotrophic tyrosine kinase, receptor, type 3	6.347559	5.990314	-0.4
Pcna	proliferating cell nuclear antigen	10.28271	10.64208	0.4
Mki67	antigen identified by monoclonal antibody Ki 67	4.799019	4.799019	0.0
Ccnd1	cyclin D1	9.439362	11.09856	1.7
Cntf	ciliary neurotrophic factor receptor	10.44431	10.44431	0.0
Gdnf	glial cell line derived neurotrophic factor	6.911917	7.776315	0.9
Aqp1	aquaporin 1	9.807625	7.814171	-2.0
Fos	FBJ osteosarcoma oncogene	11.96496	12.49366	0.5
Nfkb1	nuclear factor of kappa light polypeptide gene enhancer in B cells 1, p105	8.410229	9.486856	1.1

Tnf	tumor necrosis factor	6.7052	6.7052	0.0
Ccl2	chemokine (C-C motif) ligand 2	6.402283	10.11604	3.7
Crb1	crumbs family member 1, photoreceptor morphogenesis associated	11.92124	10.88318	-1.0
Ace2	angiotensin I converting enzyme (peptidyl-dipeptidase A) 2	4.17806	4.17806	0.0
Ncan	neurocan	7.059061	7.436745	0.4
Vcan	versican	5.366831	7.601713	2.2
Bcan	brevican	6.602848	6.602848	0.0
Cd44	CD44 antigen	7.835063	10.75019	2.9
Rac1	RAS-related C3 botulinum substrate 1	10.58856	11.14308	0.6
Rac2	RAS-related C3 botulinum substrate 2	6.715644	6.64817	-0.1
Rac3	RAS-related C3 botulinum substrate 3	6.947626	7.163431	0.2
Rhoa	ras homolog gene family, member A	11.11479	11.76252	0.6
Rhob	ras homolog gene family, member B	9.169951	10.23754	0.6
Rhoc	ras homolog gene family, member C	8.911922	9.589165	1.1
Gpx1	glutathione peroxidase 1	7.744192	8.224051	0.5
Il6st	interleukin 6 signal transducer	10.98721	11.81941	0.8
Lifr	leukemia inhibitory factor receptor	9.5482	9.000968	-0.5
Cntfr	ciliary neurotrophic factor receptor	10.44431	10.44431	0.0
Stat3	signal transducer and activator of transcription 3	9.444607	11.28477	1.8

Lif	leukemia inhibitory factor	6.396519	9.344319	2.9
Mt1	metallothionein 1	10.30838	12.41494	2.1
Mt2	metallothionein 2	8.264072	12.11129	3.8
Cp	ceruloplasmin	12.69996	13.53588	0.8
Lcn2	lipocalin 2	7.960248	12.71421	4.8
Chi3l1	Chitinase 3 Like 1	9.969642	10.76591	3.7
Bag3	BCL2-associated athanogene 3	8.030416	9.648602	0.8
Bag1	BCL2-associated athanogene 1	8.359955	8.655867	1.6
S100a1	S100 calcium binding protein A1	8.86525	10.03048	0.3
Clic4	chloride intracellular channel 4 (mitochondrial)	9.118465	10.49558	1.2
Clic1	chloride intracellular channel 1	7.760585	10.41751	1.4
Hdac11	histone deacetylase 11	8.182041	7.539239	2.7

Supplementary Table 9: Fold change of 21 genes in Müller glia after LD and Dicer deletion. *genes with >2.7 (LD) and >1.4 fold increase (cKO) are in bold.

Gene	Undamaged control log2 relative expression (Microarray)	Light Damage log2 relative expression (Microarray)	Fold change light damage versus control	Wild type (RNA-Seq) log2CPM	Dicer-cKO (RNA-Seq) log2CPM	Fold change cKO versus wild type
**Maff**	**5.31757**	**8.7607**	**3.44**	**6.18847**	**8.76958**	**2.58**
**Atf3**	**8.04569**	**11.1855**	**3.14**	**6.83319**	**8.98196**	**2.15**
Irf1	7.89435	9.26844	1.37	6.65689	8.79824	2.14
Arc	7.99967	9.42568	1.43	6.93241	8.75483	1.82
**Egr2**	**7.16072**	**9.94098**	**2.78**	**7.02422**	**8.8231**	**1.80**
Rrs1	7.41351	8.87402	1.46	4.62288	6.37159	1.75
**Gadd45b**	**7.56354**	**11.6491**	**4.09**	**4.78525**	**6.4322**	**1.65**
Fosl2	7.10696	8.63265	1.53	5.19051	6.54333	1.35
Csrnp1	7.04901	8.99495	1.95	6.24975	7.43475	1.19
Cebpb	7.8281	9.37884	1.55	4.73147	5.78673	1.06
Lmna	7.01591	8.57848	1.56	4.51125	5.56106	1.05
C4b	7.77481	9.44723	1.67	4.94628	5.9242	0.98
Epha2	7.82134	9.26089	1.44	5.8779	6.7657	0.89
Mt2	8.26407	12.1113	3.85	6.93133	7.81361	0.88
Nfkbia	8.25089	10.232	1.98	5.69714	6.54235	0.85
Gfap	6.28272	10.5111	4.23	4.37759	5.20687	0.83

H2-D1	8.34902	9.89489	1.55	5.82285	6.62164	0.80
Myh9	8.34219	9.65764	1.32	6.46277	7.24567	0.78
Hbegf	8.97967	9.60301	2.81	4.24778	5.01641	0.77
Nr4a1	7.97081	10.7798	1.49	9.49231	10.2245	0.73
Sbno2	6.99263	9.6866	2.69	5.40557	6.11851	0.71
